# Supplementary material for: Non-covalent inhibitors of thioredoxin glutathione reductase with schistosomicidal activity in vivo
Source: Nat Commun. 2023 Jun 22;14:3737. doi: 10.1038/s41467-023-39444-y (PMC10287695; doi:10.1038/s41467-023-39444-y)
Supplement: Supplementary file 1 — Supplementary Information [file 41467_2023_39444_MOESM1_ESM.pdf]

# Supplementary Information

Table of Contents

*1 Supplementary Methods* ........ *2*

**1.1 Experimental: Synthesis of 1-12 and PRP** ........ **2**

**1.2 HPLC data for 1-12 and PRP** ........ **22**

*2 Supplementary Figures and Tables* ........ *31*

*3 Supplementary References* ........ *46*

# 1 Supplementary Methods

## 1.1 Experimental: Synthesis of 1-12 and PRP

**Supplementary Table 1.** Compound numbers in the main manuscript and the corresponding laboratory codes. The numbering of compounds in the synthetic part of the supplemental material is different from that in the main manuscript and the remainder of the Supplemental Figure and Tables and is applicable to the synthesis section only. All the original laboratory notebook compound names for the final compounds were kept to maintain scientific rigor and reproducibility.

| ID[a] | Code[b] | Code2[c] | MW[d] | logP[e] | donors[f] | acceptors[g] | druglike[i] |
|---|---|---|---|---|---|---|---|
| **1** | 8VP70 | 10VP28 | 368.47699 | 3.7992406 | 1 | 4 | 1 |
| **2** | 8VP101 | 9VP21 | 352.522 | 5.4329996 | 1 | 2 | 1 |
| **3** | 8VP83 | 10VP76 | 324.46799 | 4.4308457 | 1 | 2 | 1 |
| **4** | 8VP121-1 | 9VP105 | 330.51599 | 4.316957 | 1 | 2 | 1 |
| **5** | 8VP131 | | 334.46298 | 4.8721457 | 1 | 2 | 1 |
| **6** | 8VP192 | 10VP30 | 362.517 | 4.964715 | 0 | 2 | 1 |
| **7** | 9VP108 | 9VP196 | 344.49899 | 4.396966 | 1 | 2 | 1 |
| **8** | 9VP173 | 10VP24 | 379.59198 | 5.1738877 | 1 | 2 | 1 |
| **9** | 9VP128-2 | 10VP107 | 414.54599 | 2.8294728 | 4 | 5 | 1 |
| **10** | 10VP91 | | 393.57498 | 4.693522 | 1 | 2 | 1 |
| **11** | 8VP71 | | 309.457 | 3.9064167 | 3 | 3 | 1 |
| **12** | 9VP51 | | 297.44598 | 3.6211317 | 1 | 2 | 1 |
| **PRP** | 9VP40 | | | ND[j] | ND[j] | ND[j] | ND[j] |

[a)] - compound number in the main manuscript; [b)] - original laboratory code; [c)] – secondary code when compound was resynthesized; [d)] – molecular weight; [e)] – logP value calculated in MOE;[1] [f)] – number of hydrogen bond donors calculated in MOE;[1] [g)] - number of hydrogen bond acceptors calculated in MOE;[1] [i)] Lipinski et al "oral activity"/bioavailability druglikeness[2] criterion calculated in MOE.[1] The of value of 1 means "druglike/orally active"; [j)] – not determined/calculated.

All reactions were carried out under inert atmosphere of nitrogen and monitored by thin-layer chromatography with silica gel 60 $F_{254}$ precoated glass plates. Visualization of TLC plates was performed by UV light irradiation (254 nm) or staining with phosphomolybdic acid. All reagents were purchased from commercial suppliers and were used without further purification. Chromatographic purifications were performed using an HPFC Biotage Isolera™ Four 3.0 system using prepacked flash chromatography cartridges in normal phase (irregular silica, 40-60 µm; hexanes/ethyl acetate gradient) or reverse phase (Biotage KP-C18-HS, water/methanol gradient) modes with UV detection at 254 and 280 nm. $^{1}$H NMR spectra were recorded on Bruker spectrometer at 400 MHz. $^{13}$C NMR spectra were recorded on Bruker spectrometer at 100 MHz. Chemical shifts were reported in parts per million (ppm) and calibrated with $CDCl_3$ residual peak. Coupling constants were reported in Hz and the standard abbreviations indicating multiplicity were used as follows: s = singlet, bs = broad singlet, d = doublet, t = triplet, q = quartet, and m = multiplet. Chromatographic purities of the final compounds were determined using a Shimadzu HPLC system equipped with CN-propyl column (NUCLEODUR 100-3 CN-RP, 2×50 mm, 3 µm) utilizing water/acetonitrile=90/10+0.1% formic acid eluent system for phase A and methanol+0.1% formic acid for phase B. Purities of all final products were found to be superior to 95% and determined by integration of the chromatogram after subtraction of the background using Labsolutions LCMS software at wavelengths giving the maximum absorbance. High-resolution mass-spectra (HRMS) were performed on Waters Synapt G2-Si ESI/LCMS instrument. Optical rotation was measured on Rudolph Research Analytical AUTOPOL IV automatic polarimeter.

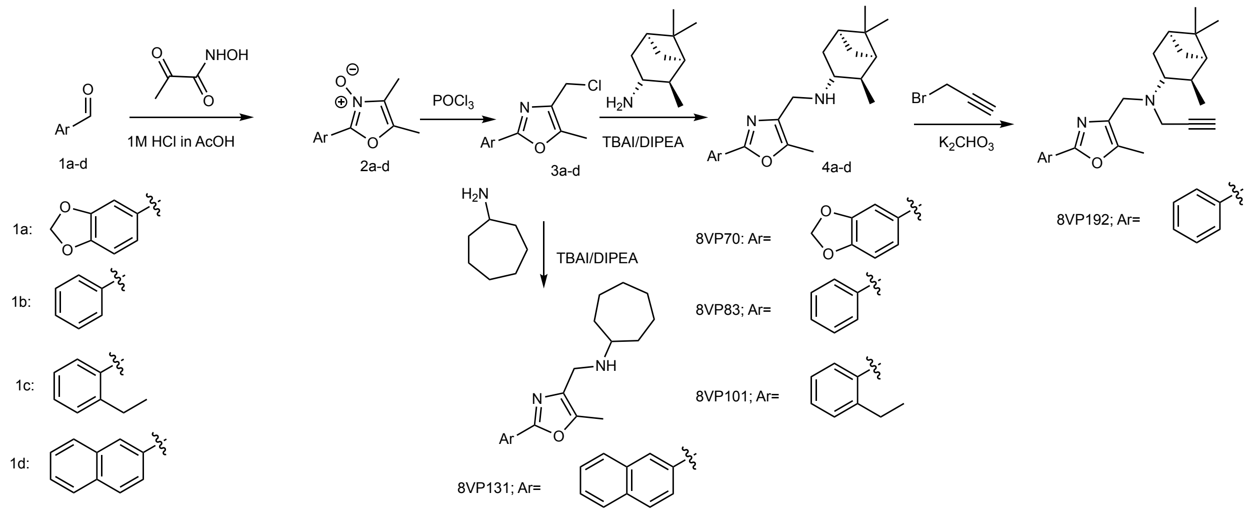


**Supplementary Figure 1.** Synthesis of 8VP70, 8VP83, 8VP101, and 8VP192.

**General procedure A: synthesis of oxazole N-oxides 2a-d.** Oxazole N-oxides were assembled from commercial benzaldehydes (1a-d) and butane-2,3-dione monoxime similarly to the reported procedure.[3]

1M HCl in acetic acid (2 eq) was added to the mixture of aryl aldehyde (1 eq) and 2,3-butanedione monoxime (1.1 eq) under cooling with ice-water. The resultant mixture was stirred for 12 hrs at ambient temperature. Diethyl ether was then added to the reaction to precipitate the product and the resultant slurry was stirred for 30 min. The precipitate was filtered and washed with ether three times. The cake was suspended in methylene chloride/water and conc. $NH_4OH$ was added to adjust pH of aqueous layer to 8. The resulting mixture was stirred for 20 min and aqueous layer was removed. Organic phase was washed with brine, dried over $Na_2SO_4$ and the solvent was removed *in vacuo* to provide target oxazole N-oxides with sufficient purity. If necessary, product can be purified on silica gel using $CH_2Cl_2$/MeOH=9/1 eluent mixture.

**2-(Benzo[d][1,3]dioxol-5-yl)-4,5-dimethyloxazole 3-oxide (2a):** Synthesized according to general method A from piperonal aldehyde. Yellow powder, yield 95%. 1H NMR (400 MHz): δ

8.10 (dd, $J_1$=8.32 Hz, $J_2$=1.65 Hz, 1H), 7.98 (d, $J$=1.56 Hz, 1H), 6.92 (d, $J$=8.31 Hz, 1H), 6.03 (s, 2H), 2.34 (d, $J$=0.76 Hz, 3H), 2.19 (d, $J$=0.72 Hz, 3H). $^{13}$C NMR (400 MHz, $CDCl_3$): δ 149.1, 147.8, 146.2, 141.0, 128.9, 120.0, 117.5, 108.6, 105.1, 101.5, 11.0, 6.3. HRMS calcd for $C_{12}H_{12}NO_4$ $[M+H]^+$ 234.0766, found 234.0769.

**4,5-Dimethyl-2-phenyloxazole 3-oxide (2b[4]):** Synthesized according to general method A from benzaldehyde. Off-white powder, yield 82%. 1H NMR (400 MHz): δ 8.44 (d, $J$=7.68 Hz, 2H), 7.52-7.36 (m, 3H), 2.34 (s, 3H), 2.20 (s, 3H).

**2-(2-Ethylphenyl)-4,5-dimethyloxazole 3-oxide (2c[5]):** Synthesized according to general method A from 2-ethylbenzaldehyde. Colorless oil, yield 62%. 1H NMR (400 MHz): δ 7.65 (dd, $J_1$=7.87 Hz, $J_2$=1.27 Hz, 1H), 7.49 (dt, $J_1$=7.54 Hz, $J_2$=1.39 Hz, 1H), 7.35 (d, $J$=7.75 Hz, 1H), 3.08 (q, $J$=7.44 Hz, 2H), 2.52 (s, 3H), 2.08 (s, 3H), 1.29 (t, $J$=1.50 Hz, 3H).

**4,5-Dimethyl-2-(naphthalen-2-yl)oxazole 3-oxide (2d[6]):** Synthesized according to general method A from naphthaldehyde. Off-white powder, yield 45%. 1H NMR (400 MHz): δ 9.33 (s, 1H), 8.26 (dd, $J_1$=8.74 Hz, $J_2$=1.62 Hz, 1H), 7.99-7.96 (m, 1H), 7.93 (d, $J$=8.80 Hz, 1H), 7.87-7.82 (m, 1H), 7.56-7.50 (m, 2H), 2.41 (d, $J$=0.68 Hz, 3H), 2.26 (d, $J$=0.68 Hz, 3H).

**General procedure B: synthesis of 2-Aryl-4-(chloromethyl)-5-methyloxazoles (3a-d).**
Synthesis of compounds **3a-d** was performed according to the published literature method.[3] $POCl_3$ (1.1 eq) was added dropwise to a solution of oxazole N-Oxide (2a-d) in anhydrous chloroform (5 ml/mmol) and the resulting mixture was refluxed for 30 min. After cooling reaction mixture was treated with ice/$NH_4OH$ (pH=8) and aqueous layer was extracted with methylene chloride (three times). Combined organic layers were washed with brine, dried over

$Na_2SO_4$ and the solvent was evaporated. The residue was purified by flash chromatography on silica gel using hexanes/ethyl acetate mixture to afford the desired compounds.

**2-(Benzo[d][1,3]dioxol-5-yl)-4-(chloromethyl)-5-methyloxazole (3a):** Synthesized according to general method B from compound **2a**. White powder, 73%. $^{1}$H NMR (400 MHz, $CDCl_3$): δ 7.55 (dd, $J_1$=8.17 Hz, $J_2$=1.69 Hz, 1H), 7.46 (d, $J$=1.62 Hz, 1H), 6.86 (d, $J$=8.09 Hz, 1H), 6.02 (s, 2H), 4.53 (s, 2H), 2.40 (s, 3H). $^{13}$C NMR (400 MHz, $CDCl_3$): δ 159.9, 149.5, 148.1, 146.1, 132.7, 121.4, 120.9, 108.5, 106.6, 101.5, 37.3, 10.3. HRMS calcd for $C_{12}H_{11}ClNO_3$ $[M+H]^+$ 252.0427, found 252.0428.

**4-(Chloromethyl)-5-methyl-2-phenyloxazole (3b[4]):** Synthesized according to general method B from compound 2b. White solids, 95%. $^{1}$H NMR (400 MHz, $CDCl_3$): δ 8.03-7.98 (m, 2H), 7.47-7.41 (m, 3H), 4.56 (s, 2H), 2.43 (s, 3H).

**4-(Chloromethyl)-2-(2-ethylphenyl)-5-methyloxazole (3c[5]):** Synthesized according to general method B from compound 2c. Colorless oil, 58%. $^{1}$H NMR (400 MHz): δ 7.88 (d, $J$=7.78 Hz, 1H), 7.39-7.22 (m, 3H), 4.46 (s, 2H), 3.08 (q, $J$=7.49 Hz, 2H), 2.40 (s, 3H), 1.24 (t, $J$=7.49 Hz, 3H).

**4-(Chloromethyl)-5-methyl-2-(naphthalen-2-yl)oxazole (3d[6]):** Synthesized according to general method B from compound 2d. White solids, 77%. $^{1}$H NMR (400 MHz): δ 8.51 (s, 1H), 8.10 (dd, $J_1$=8.59 Hz, $J_2$=1.69 Hz, 1H), 7.94-7.83 (m, 3H), 7.56-7.49 (m, 2H), 4.59 (s, 2H), 2.48 (s, 3H).

**General procedure C: synthesis of N-((2-aryl-5-methyloxazol-4-yl)methyl)amines (8VP70, 8VP83,8VP101, 8VP131).** To the solution of amine (2 eq) in methylene chloride (5ml/mmol) were added diisopropylethylamine (1.2 eq) and tetrabutylammonium iodide (0.05 eq). The

reaction mixture was cooled down with ice-water bath and the solution of 2-aryl-4-(chloromethyl)-5-methyloxazole (1 eq) in methylene chloride (5ml/mmol) was added dropwise. The resulting mixture was stirred at ambient temperature for 12 hrs, then washed with water, brine and dried over $Na_2SO_4$. Solvent was evaporated and the residue was purified on reverse phase Biotage KP-C18 cartridge (water/methanol eluent) to afford the final compound.

**(1R,2R,3R,5S)-N-((2-(Benzo[d][1,3]dioxol-5-yl)-5-methyloxazol-4-yl)methyl)-2,6,6-trimethylbicyclo[3.1.1]heptan-3-amine (8VP70):** Synthesized according to general method C from compound 3a and (1R,2R,3R,5S)-(-)-isopinocampheylamine. White solidified oil, 48%. $^1H$ NMR (400 MHz, $CDCl_3$): δ 7.53 (dd, $J_1$=8.12 Hz, $J_2$=1.62 Hz, 1H), 7.45 (d, $J$=1.53 Hz), 6.85 (d, $J$=8.11 Hz, 1H), 6.01 (s, 2H), 3.65 (AB system, $J_{AB}$=13.29 Hz, 2H), 2.93-2.87 (m, 1H), 2.42-2.28 (m, 5H), 1.99-1.93 (m, 1H), 1.89-1.77 (m, 2H), 1.73-1.66 (m, 1H), 1.21 (s, 3H), 1.09 (d, $J$=7.26 Hz, 3H), 1.02 (d, $J$=9.58 Hz, 1H), 0.97 (s, 3H). $^{13}C$ NMR (400 MHz, $CDCl_3$): δ 159.5, 149.0, 147.9, 144.0, 134.4, 122.2, 120.6, 108.5, 106.5, 101.4, 56.2, 47.9, 44.8, 42.8, 41.8, 38.6, 36.4, 33.6, 27.8, 23.4, 21.5. HRMS calcd for $C_{22}H_{29}N_2O_3$ $[M+H]^+$ 369.2178, found 369.2170. $\alpha_{589}^{23}$−59 (c 0.71, $CHCl_3$).

**(1R,2R,3R,5S)-2,6,6-Trimethyl-N-((5-methyl-2-phenyloxazol-4-yl)methyl)bicyclo[3.1.1]heptan-3-amine (8VP83):** Synthesized according to general method C from compound 3b and (1R,2R,3R,5S)-(-)-isopinocampheylamine. Colorless oil, 70%. $^1H$ NMR (400 MHz, $CDCl_3$): δ 7.99-7.94 (m, 2H), 7.44-7.35 (m, 3H), 3.70 (AB system, $J_{AB}$=13.25 Hz, 2H), 3.00-2.92 (m, 2H), 2.43-2.26 (m, 5H), 1.99-1.84 (m, 2H), 1.80-1.69 (m, 2H), 1.20 (s, 3H), 1.09 (d, $J$=7.16 Hz, 3H), 1.05 (d, $J$=9.52 Hz, 1H), 0.95 (s, 3H). $^{13}C$ NMR (400 MHz, $CDCl_3$): δ 159.7, 144.6, 134.3, 129.7, 128.6, 127.7, 125.9, 56.1, 47.9, 44.6, 42.6, 41.7, 38.6, 36.1, 33.5,

27.8, 23.4, 21.4, 10.3. HRMS calcd for $C_{21}H_{29}N_2O$ $[M+H]^+$ 325.2280, found 325.2275. $\alpha_{589}^{23}$−61 (c 1.23, $CHCl_3$).

**(1R,2R,3R,5S)-N-((2-(2-Ethylphenyl)-5-methyloxazol-4-yl)methyl)-2,6,6-trimethylbicyclo[3.1.1]heptan-3-amine (8VP101):** Synthesized according to general method C from compound 3c and (1R,2R,3R,5S)-(-)-isopinocampheylamine. Colorless oil, 59%. $^1$H NMR (400 MHz, $CDCl_3$): δ 7.86 (d, *J*=7.72 Hz, 1H), 7.37-7.19 (3H), 3.69 (AB system, $J_{AB}$=13.24 Hz, 2H), 3.07 (q, *J*=7.43 Hz, 2H), 2.98-2.90 (m, 1H), 2.42-2.29 (m, 5H), 2.00-1.93 (m, 1H), 1.91-1.66 (m, 4H), 1.27-1.18 (m, 6H), 1.09 (d, *J*=6.84 Hz, 3H), 1.03 (d, *J*=9.56 Hz, 1H), 0.96 (s, 3H). $^{13}$C NMR (400 MHz, $CDCl_3$): δ 159.8, 143.9, 143.2, 134.3, 129.7, 129.6, 128.9, 126.4, 125.7, 55.9, 47.9, 44.7, 42.6, 41.7, 38.5, 36.3, 33.5, 27.8, 27.2, 23.3, 21.4, 15.4, 10.2. HRMS calcd for $C_{23}H_{33}N_2O$ $[M+H]^+$ 353.2593, found 353.2593. $\alpha_{589}^{23}$−55 (c 1.1, $CHCl_3$).

**N-((5-Methyl-2-(naphthalen-2-yl)oxazol-4-yl)methyl)cycloheptanamine (8VP131).** Synthesized according to general method C from compound 3c and cycloheptylamine. Solidified yellow oil, 59%. $^1$H NMR (400 MHz, $CDCl_3$): δ 8.46 (d, J=0.89 Hz, 1H), 8.07 (dd, $J_1$=8.57 Hz, $J_2$=1.70 Hz, 1H), 7.92-7.79 (m, 3H), 7.52-7.46 (m, 2H), 3.68 (s, 2H), 2.75-2.67 (m, 1H), 2.39 (s, 3H), 1.92-1.85 (m, 2H), 1.72-1.62 (m, 2H), 1.58-1.35 (m, 8H). $^{13}$C NMR (400 MHz, $CDCl_3$): δ 159.9, 145.1, 134.1, 133.9, 133.0, 128.5, 128.4, 127.8, 126.9, 126.6, 125.6, 124.9, 123.2, 58.3, 41.8, 34.3, 28.2, 24.4, 10.4. HRMS calcd for $C_{22}H_{27}N_2O$ $[M+H]^+$ 335.2123, found 335.2118.

**Synthesis of (1R,2R,3R,5S)-2,6,6-trimethyl-N-((5-methyl-2-phenyloxazol-4-yl)methyl)-N-(prop-2-yn-1-yl)bicyclo[3.1.1]heptan-3-amine (8VP192):** To 8VP83 (82 mg; 0.252 mmol) in 3 mL acetonitrile was added $K_2CO_3$ (104 mg; 3 eq) followed by the dropwise addition of propargyl bromide (90 mg; 3 eq) and the resulting mixture was stirred at ambient temperature for 12 hrs.

The reaction mixture was partitioned between methylene chloride and water. Aqueous layer was extracted with methylene chloride, and the combined organic layers were washed with brine, dried over $Na_2SO_4$ and concentrated under reduced pressure. The residue was purified by flash chromatography (hexanes/EtOAc=90:10) to afford the desired compound as yellow oil which solidified upon standing (77 mg; 82%). $^1$H NMR (400 MHz, $CDCl_3$): δ 7.99-7.95 (m, 2H), 7.42-7.34 (m, 3H), 3.73 (AB system, $J_{AB}$=14.07 Hz, 2H), 3.52-3.41 (m, 2H), 3.40-3.32 (m, 1H), 2.42 (s, 3H), 2.33-2.25 (m, 1H), 2.22-2.13 (m, 2H), 2.12-2.03 (m, 1H), 2.01-1.93 (m, 2H), 1.83-1.78 (m, 1H), 1.19 (s, 3H), 1.11 (d, $J$=6.96 Hz, 3H), 0.99 (s, 3H). $^{13}$C NMR (400 MHz, $CDCl_3$): δ 159.4, 145.9, 133.7, 128.5, 127.8, 81.5, 72.4, 60.2, 48.2, 45.1, 41.7, 40.8, 38.9, 33.4, 28.9, 28.0, 23.4, 21.6, 10.5. HRMS calcd for $C_{24}H_{31}N_2O$ $[M+H]^+$ 363.2436, found 363.2430. $\alpha_{589}^{23}-56$ (c 0.97, $CHCl_3$).

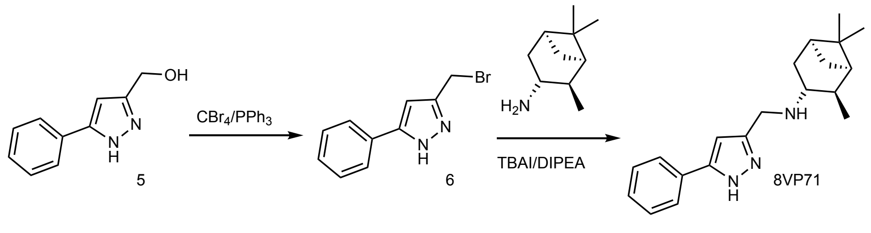


**Supplementary Figure 2.** Synthesis of 8VP71.

**(5-Phenyl-1*H*-pyrazole-3-yl) methanol (5)**. Compound **5** was synthesized according to the literature method.[7] White solids, 51 %. $^1$H NMR (400 MHz, $CDCl_3$): δ 12.99, 12.72 (total 1H, each br s), 7.81-7.68 (m, 2H), 7.46-7.21 (m, 3H), 6.62-6.52 (m, 1H), 5.29-4.94 (m, 1H), 4.53-4.38 (m, 2H).

**General procedure D: synthesis of 3-(bromomethyl)-5-phenyl-1*H*-pyrazole (6[8]).** To compound **5** (100 mg; 0.57 mmol) in 3 mL methylene chloride under cooling with ice-water bath was added $CBr_4$ (286 mg; 0.86 mmol) followed by portion wise addition of triphenyl phosphine

(226 mg; 0.86 mmol). Reaction mixture was stirred at ambient temperature for 12 hrs and then solvent was removed in vacuo. The crude residue was purified by flash chromatography (hexanes/EtOAc=90:10) to afford previously reported compound **6** as white solids. (47 mg; 36%). $^{1}$H NMR (400 MHz, $CDCl_3$): δ 8.32 (s, 1H), 7.63-7.58 (m, 2H), 7.44-7.31 (m, 3H), 6.59 (s, 1H), 4.51 (s, 2H).

**(1R,2R,3R,5S)-2,6,6-trimethyl-N-((5-phenyl-1*H*-pyrazol-3-yl)methyl)bicyclo[3.1.1]heptan-3-amine (8VP71).** Synthesized according to general method C from compound **6** and (1R,2R,3R,5S)-(-)-isopinocampheylamine. Colorless oil, 45%. $^{1}$H NMR (400 MHz, $CDCl_3$): δ 7.74 (d, *J*=7.53 Hz, 2H), 7.40 (t, *J*=7.53 Hz, 2H), 7.32 (m, 1H), 6.48 (s, 1H), 3.93 (AB system, $J_{AB}$=14.12 Hz, 2H), 2.97-2.88 (m, 1H), 2.46-2.28 (m, 2H), 2.00-1.92 (m, 1H), 1.89-1.76 (m, 2H), 1.69-1.59 (m, 1H), 1.21 (s, 3H), 1.11 (d, *J*=7.11 Hz), 3H), 0.94 (s, 3H). $^{13}$C NMR (400 MHz, $CDCl_3$): δ 149.7, 146.1, 132.6, 128.6, 127.7, 125.6, 56.4, 47.9, 45.0, 43.4, 41.7, 38.5, 36.5, 33.8, 27.7, 23.4, 21.5. HRMS calcd for $C_{20}H_{28}N_3$ $[M+H]^+$ 310.2283, found 310.2289. $\alpha_{589}^{23}$–28 (c 1.19, $CHCl_3$).

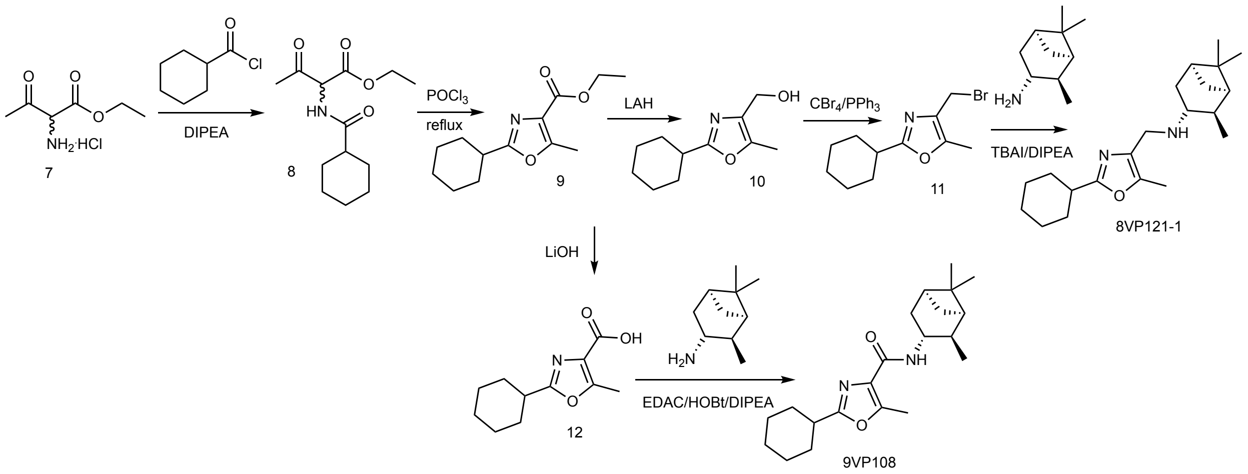


**Supplementary Figure 3.** Synthesis of 8VP121-1 and 9VP108.

**(2-Cyclohexyl-5-methyloxazol-4-yl)-methanol (10)**. Compound **10** was synthesized according to a literature method.[9] Yellowish oil, 90 %. $^1$H NMR (400 MHz, $CDCl_3$): δ 4.46 (s, 2H), 2.72-2.64 (m, 1H), 2.26 (s, 3H), 2.04-1.96 (m, 2H), 1.82-1.61 (m, 4H), 1.57-1.45 (m, 2H), 1.40-1.13 (m, 2H).

**4-(Bromomethyl)-2-cyclohexyl-5-methyloxazole (11)**. Synthesized according to general procedure D from compound **10**. Colorless oil, 55%. $^1$H NMR (400 MHz, $CDCl_3$): δ 4.34 (s, 2H), 2.73-2.64 (m, 1H), 2.25 (s, 3H), 2.05-1.98 (m, 2H), 1.82-1.75 (m, 2H), 1.70-1.64 (m, 1H), 1.57-1.47 (m, 2H), 1.38-1.18 (m, 3H). $^{13}$C NMR (400 MHz, $CDCl_3$): δ 166.4, 145.3, 131.0, 37.4, 30.5, 25.7, 25.6, 24.2, 10.2. HRMS calcd for $C_{11}H_{17}BrNO$ $[M+H]^+$ 258.0494, found 258.0497.

**(1R,2R,3R,5S)-N-((2-Cyclohexyl-5-methyloxazol-4-yl)methyl)-2,6,6-trimethylbicyclo[3.1.1]heptan-3-amine (8VP121-1).** Synthesized according to general method C from compound **11** and (1R,2R,3R,5S)-(-)-isopinocampheylamine. Colorless oil, 64%. $^1$H NMR (400 MHz, $CDCl_3$): δ 3.54 (AB system, $J_{AB}$=12.99 Hz, 2H), 2.86-2.79 (m, 1H), 2.72-2.63 (m, 1H), 2.36-2.24 (m, 2H), 2.23 (s, 3H), 2.02-1.89 (m, 3H), 1.85-1.74 (m, 3H), 1.70-1.59 (m, 3H), 1.57-1.45 (m, 2H), 1.38-1.21 (m, 3H), 1.19 (s, 3H), 1.05 (d, $J$=7.24 Hz, 3H), 0.99 (d, $J$=9.68 Hz, 1H), 0.94 (s, 3H). $^{13}$C NMR (400 MHz, $CDCl_3$): δ 166.2, 143.5, 131.9, 56.0, 47.9, 44.4, 43.4, 42.5, 41.7, 38.6, 37.5, 35.9, 33.4, 30.7, 30.6, 27.8, 25.8, 25.7, 23.4, 21.4, 10.1. HRMS calcd for $C_{21}H_{35}N_2O$ $[M+H]^+$ 331.2749, found 331.2745. $\alpha_{589}^{23}$–41 (c 0.80, $CHCl_3$).

**2-Cyclohexyl-5-methyloxazole-4-carboxylic acid (12).** Compound **12** was synthesized according to literature method.[10] Off-white solids, 90 %. $^1$H NMR (400 MHz, $CDCl_3$): δ 2.78-2.70 (m, 1H), 2.58 (s, 3H), 2.07-1.97 (m, 2H), 1.84-1.63 (m, 3H), 1.61-1.49 (m, 2H), 1.41-1.19 (m, 3H).

**General procedure E: 2-cyclohexyl-5-methyl-N-((1R,2R,3R,5S)-2,6,6-trimethylbicyclo[3.1.1]heptan-3-yl)oxazole-4-carboxamide (9VP108).** To compound **12** (155 mg; 0.74 mmol) in 6 mL methylene chloride under cooling with ice-water bath was added 1-hydroxybenzotriazole (120 mg; 0.89 mmol), followed by the addition of 1-ethyl-3-(3-dimethylaminopropyl)carbodiimide hydrochloride (170 mg; 0.89 mmol). After 10 min of stirring (1R,2R,3R,5S)-(-)-isopinocampheylamine (150 mg; 0.98 mmol) and diisopropylethylamine amine (115 mg; 0.89 mmol) were added and the resulting mixture was stirred at ambient temperature for 12 hrs. Then reaction mixture was diluted with methylene chloride/water=5mL/5mL and pH of an aqueous layer was adjusted to 3 with 1M HCl. Aqueous layer was extracted with methylene chloride three times. Combined organic layers were washed with brine, dried over $Na_2SO_4$ and concentrated. The residue was purified by flash chromatography (hexanes/EtOAc=90:10) to afford the desired compound as solidified white oil (208 mg; 82%). $^1$H NMR (400 MHz, $CDCl_3$): δ 6.79 (d, *J*=9.16 Hz, 1H), 4.44-4.35 (m, 1H), 2.75-2.59 m, 2H), 2.59 (s, 3H), 2.45-2.37 (m, 1H), 2.04-1.86 (m, 3H), 1.85-1.76 (m, 2H), 1.72-1.47 (m, 7H), 1.41-1.23 (m, 2H), 1.22 (s, 3H), 1.12 (d, *J*=7.08 Hz, 3H), 1.07 (s, 3H), 0.95 (d, *J*=9.75 Hz, 1H). $^{13}$C NMR (400 MHz, $CDCl_3$): δ 164.9, 161.8, 151.9, 128.7, 47.8, 47.1, 45.9, 41.6, 38.5, 37.4, 37.1, 35.2, 30.5, 28.1, 25.7, 25.5, 23.3, 20.7, 11.5. HRMS calcd for $C_{21}H_{33}N_2O_2$ $[M+H]^+$ 345.2542, found 345.2538. $\alpha_{589}^{23}$−18 (c 1.18, $CHCl_3$).

**(1R,2R,3R,5S)-2,6,6-trimethyl-*N*-((1-methyl-1*H*-benzo[*d*]imidazol-2-yl)methyl)bicyclo[3.1.1]heptan-3-amine (9VP51)** was synthesized according to the reported procedure.[11] Solidified white oil, 38 %. $^1$H NMR (400 MHz, $CDCl_3$): δ 7.73-7.68 (m, 1H), 7.33-7.29 (m, 1H), 7.27-7.19 (m, 2H), 4.07 (AB system, $J_{AB}$=13.62 Hz, 2H), 3.84 (s, 3H), 3.00-2.93 (m, 1H), 2.48-2.39 (m, 1H), 2.36-2.28 (m, 1H), 1.99-1.93 (m, 1H), 1.88-1.77 (m 2H), 1.74-1.66

(m 1H), 1.20 (s, 3H), 1.11 (d, *J*=7.23 Hz, 3H), 0.98-0.93 (m, 4H). $^{13}C$ NMR (400 MHz, $CDCl_3$): δ 153.0, 142.3, 136.1, 122.4, 121.8, 119.4, 109.0, 57.3, 47.8, 45.0, 44.8, 41.7, 38.5, 36.3, 33.7, 29.9, 27.8, 23.4, 21.5. HRMS calcd for $C_{19}H_{28}N_3$ $[M+H]^+$ 298.2283, found 298.2283. $\alpha_{589}^{23}$−47 (c 1.25, $CHCl_3$).

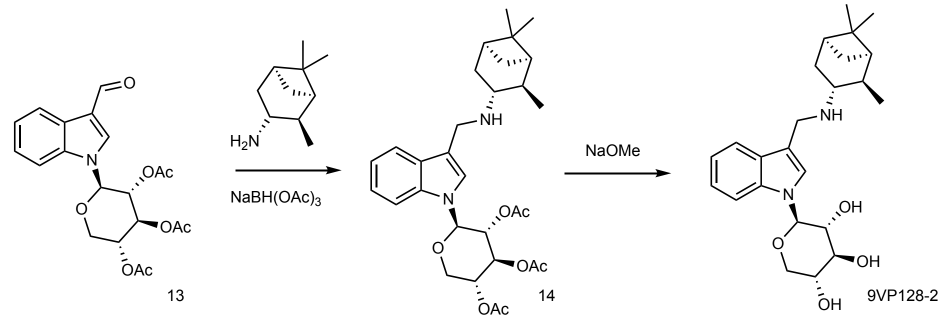


**Supplementary Figure 4.** Synthesis of 9VP128-2.

**(2S,3R,4S,5R)-2-(3-formyl-1*H*-indol-1-yl)tetrahydro-2*H*-pyran-3,4,5-triyl triacetate (13).** Compound **13** was synthesized according to reported literature method.[12] Yellow oil, 56 %. $^1H$ NMR (400 MHz, $CDCl_3$): δ 10.01 (s, 1H), 8.30-8.26 (m, 1H), 7.86 (s, 1H), 7.45-7.41 (m, 1H), 7.37-7.28 (m, 2H), 5.56 (d, *J*=8.68 Hz, 1H), 5.49-5.38 (m, 2H), 5.24-5.16 (m, 1H), 4.33 (dd, $J_1$=11.78 Hz, $J_2$=5.74 Hz, 1H), 3.61 (t, *J*=11.20 Hz, 1H), 2.94 (s, 3H), 2.86 (s, 3H), 2.04 (s, 3H).

**(2S,3R,4S,5R)-2-(3-((((1R,2R,3R,5S)-2,6,6-trimethylbicyclo[3.1.1]heptan-3-yl)amino)methyl)-1*H*-indol-1-yl)tetrahydro-2*H*-pyran-3,4,5-triyl triacetate (14).** To compound **13** (80 mg; 0.198 mmol) in 3 mL methylene chloride was added (1R,2R,3R,5S)-(-)-isopinocampheylamine (36 mg; 0.223 mmol) and 4A powdered sieves (100 mg). After 10 min stirring sodium triacetoxyborohydride (84 mg; 0.398 mmol) was added and the resulting mixture was stirred at ambient temperature for 12 hrs. Then reaction mixture was diluted with 6 mL

methylene chloride and washed with saturated $NaHCO_3$ solution, brine, dried over $Na_2SO_4$ and concentrated. The residue was purified by flash chromatography (EtOAc/MeOH=90/10) to afford the desired compound as colorless oil (82 mg; 77%). $^1$H NMR (400 MHz, $CDCl_3$): δ 7.66 (d, *J*=7.88 Hz, 1H), 7.36 (d, *J*=7.92 Hz, 1H), 7.25-7.19 (m, 1H), 7.18 (s, 1H), 7.15-7.10 (m, 1H), 5.53-5.39 (m, 3H), 5.20-5.12 (m, 1H), 4.24 (dd, $J_1$=11.56 Hz, $J_2$=5.76 Hz, 1H), 3.94 (AB system, $J_{AB}$=13.41 Hz, 2H), 3.56 (t, *J*=11.04 Hz, 1H), 2.98-2.90 (m, 1H), 2.43-2.26 (m, 2H), 2.06 (s, 3H), 2.03 (s, 3H), 1.98-1.92 (m, 1H), 1.88-1.79 (m, 1H), 1.79-1.74 (m, 1H), 1.74-1.67 (m, 1H), 1.66 (s, 3H), 1.19 (s, 3H), 1.05-0.98 (m, 4H), 0.94 (s, 3H). $^{13}$C NMR (400 MHz, $CDCl_3$): δ 170.2, 169.8, 168.8, 136.9, 128.4, 122.9, 122.6, 120.6, 119.6, 116.0, 109.6, 83.7, 73.0, 70.6, 68.9, 65.5, 56.0, 47.9, 44.6, 42.4, 41.7, 38.6, 35.9, 33.6, 27.8, 23.5, 21.4, 20.7, 20.2. HRMS calcd for $C_{30}H_{41}N_2O_7$ $[M+H]^+$ 541.2914, found 541.2914. $\alpha_{589}^{23}$–38 (c 1.60, $CHCl_3$).

**(2S,3R,4S,5R)-2-(3-((((1R,2R,3R,5S)-2,6,6-trimethylbicyclo[3.1.1]heptan-3-yl)amino)methyl)-1*H*-indol-1-yl)tetrahydro-2*H*-pyran-3,4,5-triol (9VP128-2).**

To **14** (60 mg; 0.111 mmol) in MeOH/DCM=1mL/0.5mL under cooling with ice-water bath was added 1M NaOMe (0.4 mL; 3.6 eq). The mixture was slowly warmed up to room temperature and neutralized with saturated solution of $NH_4Cl$ after 2 hours. The mixture was concentrated under reduced pressure. The residue was purified by reverse phase chromatography in water/MeOH=90/10-0/100 gradient to provide the desired compound as white solids. (27 mg; 59%). %). $^1$H NMR (400 MHz, $CD_3OD$): δ 7.74 (d, *J*= 7.76 Hz, 1H), 7.69 (s, 1H), 7.60 (d, *J*=8.08 Hz, 1H), 7.28 (t, *J*=7.46 Hz, 1H), 7.21 (t, *J*=7.62 Hz, 1H), 5.49 (s, 1H), 5.43 (d, *J*=9.28 Hz, 1H), 4.46 (AB system, $J_{AB}$=13.82 Hz, 2H), 3.99 (dd, $J_1$=11.22 Hz, $J_2$=5.10 Hz), 3.90 (t, *J*=8.98 Hz, 1H), 3.74-3.65 (m, 1H), 3.60-3.46 (m, 3H), 2.70-2.59 (m, 1H), 2.52-2.41 (m, 1H), 2.16-2.05 (m, 2H), 2.01-1.93 (m, 1H), 1.92-1.86 (m, 1H), 1.32-1.24 (m, 4H), 1.19-1.11 (m, 4H),

0.95 (s, 3H). $^{13}C$ NMR (400 MHz, $CD_3OD$, DEPTQ135 with quaternary carbons pulse sequence): δ peaks down (CH, $CH_3$): 128.9, 123.9, 122.0, 119.3, 112.2, 87.5, 79.1, 73.6, 71.1, 48.7, 42.5, 42.3, 27.9, 23.8, 21.1; peaks up (C, $CH_2$): 138.2, 129.0, 106.9, 69.6, 41.5, 39.8, 33.9, 32.9, 41.5, 39.8, 33.9, 32.9. HRMS calcd for $C_{24}H_{35}N2O_4$ $[M+H]^+$ 415.2597, found 415.2580. $\alpha_{589}^{23}$–43 (c 1.83, EtOH).

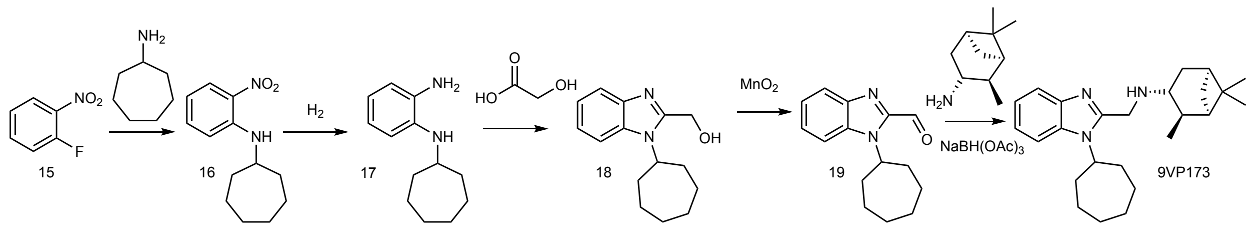


**Supplementary Figure 5.** Synthesis of 9VP173.

**1-Cycloheptyl-1H-benzo[d]imidazole-2-carbaldehyde (19).** Compound **19** was synthesized similarly to reported literature method.[13] Solidified white oil, 68 %. $^1H$ NMR (400 MHz, $CDCl_3$): δ 10.09 (s, 1H), 7.91-7.88 (m, 1H), 7.63-7.59 (m, 1H), 7.41-7.32 (m, 2H), 5.59 (m, 1H), 2.39-2.28 (m, 2H), 2.06-1.97 (m, 2H), 1.90-1.60 (m, 8H). $^{13}C$ NMR (400 MHz, $CDCl_3$): δ 185.1, 145.5, 143.5, 135.3, 126.1, 123.6, 122.5, 113.6, 58.2, 33.9, 27.4, 25.8. HRMS calcd for $C_{15}H_{19}N_2O$ $[M+H]^+$ 243.1497, found 243.1494.

**(1R,2R,3R,5S)-N-((1-Cycloheptyl-1*H*-benzo[*d*]imidazol-2-yl)methyl)-2,6,6-trimethylbicyclo[3.1.1]heptan-3-amine (9VP173).** To compound **19** (144 mg; 0.594 mmol) in 6 mL methylene chloride was added (1R,2R,3R,5S)-(-)-isopinocampheylamine (118 mg; 0.772 mmol) and 4 A powdered sieves (140 mg). After 10 min stirring sodium triacetoxyborohydride (252 mg; 1.190 mmol) was added and the resulting mixture was stirred at ambient temperature for 12 hrs. Then reaction mixture was diluted with 6 mL methylene chloride and washed with saturated $NaHCO_3$ solution, brine, dried over $Na_2SO_4$ and concentrated. The residue was purified

by flash chromatography (hexanes/EtOAc=70/30 containing 0.1% triethylamine) to afford the desired compound as colorless oil (170 mg; 76%). $^{1}$H NMR (400 MHz, $CDCl_3$): δ 7.71-7.66 (m, 1H), 7.50-7.44 (m, 1H), 7.20-7.15 (m, 2H), 4.76-4.68 (m, 1H), 4.04 (AB system, $J_{AB}$=13.36 Hz, 2H), 2.98-2.91 (m, 1H), 2.47-2.39 (m, 1H), 2.38-2.27 (m, 3H), 2.07-1.94 (m, 3H), 1.89-1.77 (m, 4H), 1.75-1.58 (m, 7H), 1.21 (s, 3H), 1.11 (d, $J$=7.21 Hz, 3H), 0.96 (s, 1H), 0.96 (d, $J$=9.32 Hz, 1H). $^{13}$C NMR (400 MHz, $CDCl_3$, DEPTQ135 with quaternary carbons pulse sequence): δ peaks down (CH, $CH_3$): 121.9, 121.4, 119.7, 58.2, 57.3, 47.9, 45.3, 41.8, 27.8, 23.4, 21.6; peaks up (C, $CH_2$): 152.3, 143.0, 133.9, 45.7, 38.6, 36.5, 33.8, 33.7, 27.6, 27.5, 26.1, 26.0. HRMS calcd for $C_{25}H_{38}N_3$ $[M+H]^+$ 380.3066, found 380.3074. $\alpha_{589}^{23}$−34 (c 1.03, $CHCl_3$).

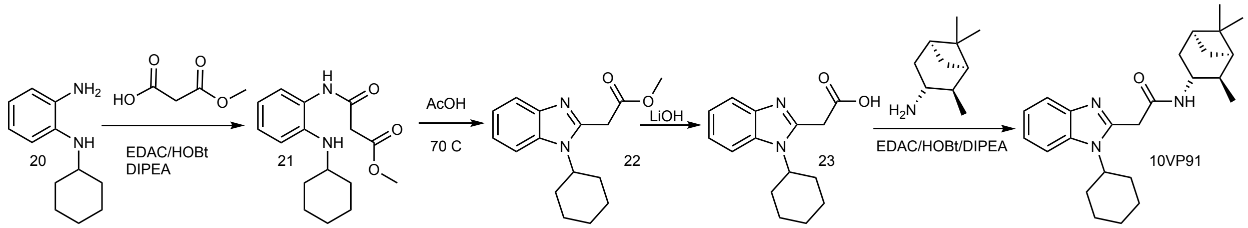


**Supplementary Figure 6.** Synthesis of 10VP91.

**Cyclohexylbenzene-1,2-diamine (20).** Compound **20** was synthesized analogously to reported literature method.[14, 15] Red solids, 95 %. $^{1}$H NMR (400 MHz, $CDCl_3$): δ 6.80-6.74 (m, 1H), 6.72-6.59 (m, 3H), 3.25-3.16 (m, 1H), 2.09-2.00 (m, 2H), 1.80-1.70 (m, 2H), 1.68-1.59 (m, 1H), 1.42-1.29 (m, 2H), 1.28-1.12 (m, 3H).

**Methyl 3-((2-(cyclohexylamino)phenyl)amino)-3-oxopropanoate (21).** Synthesized according to general method E from compound **20** and methyl hydrogen malonate. Off-white solids, 40%. Compound **21** was used in the next step without purification. $^{1}$H NMR (400 MHz, $CDCl_3$): δ 8.49 (br s, 1H), 7.37 (dd, $J_1$=7.72 Hz, $J_2$=1.28 Hz), 7.11-7.06 (m 1H), 6.81-6.70 (m, 2H), 3.79 (s,

3H), 3.50 (s, 2H), 3.26-3.17 (m, 1H), 2.06-1.97 (m, 2H), 1.80-1.70 (m, 2H), 1.67-1.57 (m, 1H), 1.41-1.15 (m, 5H).

**Methyl 2-(1-cyclohexyl-1*H*-benzo[d]imidazole-2-yl)acetate (22).** A solution of crude **21** (100 mg; 0.341 mmol) in 1.5 mL acetic acid was stirred at 70° C for 12 hrs. After cooling to room temperature, volatiles were evaporated under reduced pressure and the residue was partitioned between methylene chloride and $NaHCO_{3sat}$. Aqueous phase was extracted with methylene chloride three times and the combined organic layers were washed with brine, dried over $Na_2SO_4$ and concentrated in vacuo. The residue was purified by flash chromatography (hexanes/EtOAc=50/50-10/90) to afford the desired compound as colorless oil (84 mg; %). $^1$H NMR (400 MHz, $CDCl_3$): δ 7.73-7.68 (m, 1H), 7.58-7.53 (m, 1H), 7.23-7.17 (m, 2H), 4.18-4.08 (m, 1H), 4.05 (s, 2H), 3.71 (s, 3H), 2.29-2.16 (m, 2H), 2.00-1.89 (m, 4H), 1.83-1.75 (m, 1H), 1.49-1.27 (m, 3H). $^{13}$C NMR (400 MHz, $CDCl_3$): δ 168.9, 147.1, 143.2, 133.8, 122.2, 121.7, 120.0, 112.2, 57.1, 52.6, 35.1, 31.2, 26.1, 25.4. HRMS calcd for $C_{16}H_{21}N_2O_2$ $[M+H]^+$ 273.1603, found 273.1608.

**2-(1-Cyclohexyl-1H-benzo[d]imidazole-2-yl)-N-((1R,2R,3R,5S)-2,6,6-trimethylbicyclo[3.1.1]heptan-yl)acetamide (10VP91).** A mixture of **22** (84 mg; 0.308 mmol) and LiOH (18 mg; 0.771 mmol) in tetrahydrofuran/water=0.5 mL/0.5 mL was stirred at room temperature for 1.5 hrs. Then 2M HCl in ether (0.5 mL;1.00 mmol) was added to the reaction mixture and it was concentrated and dried in vacuo. To this residue in 3 mL methylene chloride under cooling with ice-water bath was added 1-hydroxybenzotriazole (63 mg; 0.465 mmol), followed by the addition of 1-ethyl-3-(3-dimethylaminopropyl)carbodiimide hydrochloride (89 mg; 0.465 mmol). After 10 min of stirring (1R,2R,3R,5S)-(-)-isopinocampheylamine (62 mg; 0.403 mmol) and diisopropylethylamine amine (80 mg; 0.619 mmol) were added and the

resulting mixture was stirred at ambient temperature for 12 hrs. Then reaction mixture was diluted with methylene chloride and water and pH of an aqueous layer was adjusted to 8 with $NaHCO_{3sat}$. Aqueous layer was extracted with methylene chloride three times. Combined organic layers were washed with brine, dried over $Na_2SO_4$ and concentrated. The residue was purified by flash chromatography (EtOAc, EtOAc/MeOH=90:10) to afford the desired compound as white solids (69 mg; 57%). $^1$H NMR (400 MHz, $CDCl_3$): δ 7.73-7.67 (m, 1H), 7.59-7.54 (m, 1H), 7.27-7.15 (3H), 4.39-4.29 (m, 1H), 4.28-4.19 (m, 1H), 3.91 (s, 3H), 2.56-2.47 (m, 1H), 2.38-2.29 9m, 1H), 2.28-2.13 (m, 2H), 2.00-1.85 (m, 5H), 1.84-1.69 (m, 3H), 1.55-1.41 (m, 3H), 1.38-1.27 (m, 1H), 1.18 (s, 3H), 1.03 (d, *J*=7.28 Hz, 3H), 1.00 (s, 3H), 0.82 (d, *J*=9.64 Hz, 1H). $^{13}$C NMR (400 MHz, $CDCl_3$): δ 166.0; 148.7; 142.7; 133.6; 122.1; 121.7; 119.5; 112.1; 56.5; 47.9; 47.6; 45.7; 41.4; 38.2; 36.7; 36.5; 34.8; 31.2; 27.8; 25.9; 25.2; 23.3; 20.6. HRMS calcd for $C_{25}H_{36}N_3O$ $[M+H]^+$ 394.2858, found 394.2858. $\alpha_{589}^{23}-13$ (c 0.93, $CHCl_3$).

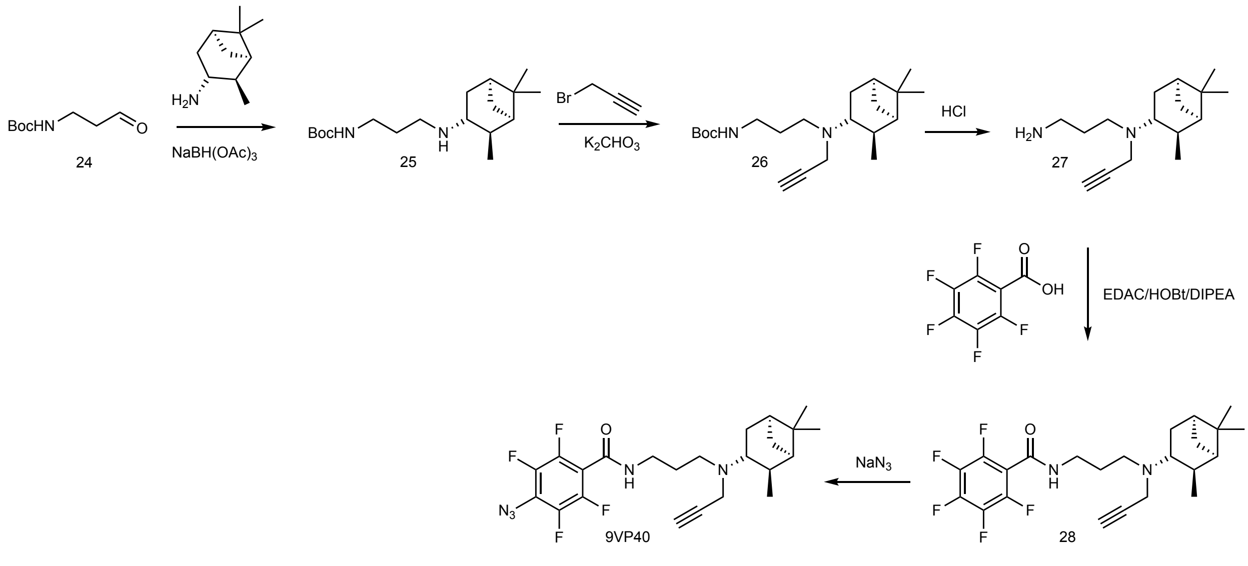


**Supplementary Figure 7.** Synthesis of 9VP40.

***tert*-Butyl (3-oxopropyl)carbamate (24).** Compound **24** was synthesized according to the literature method.[16] Colorless oil, 97%. $^1$H NMR (400 MHz, $CDCl_3$): δ 9.79 (s, 1H), 4.86 (1H, br. s), 3.42-3.37 (m, 2H), 2.68 (t, *J*=5.8 Hz, 2H), 1.45 (s, 9H).

**tert-Butyl (3-(prop-2-yn-1-yl((1R,2R,3R,5S)-2,6,6-trimethylbicyclo[3.1.1]heptan-3-yl)amino)propyl)carbamate (26).** To compound **24** (115 mg; 0.664 mmol) in 5 mL methylene chloride was added (1R,2R,3R,5S)-(-)-isopinocampheylamine (122 mg; 0.797 mmol) and 4 A powdered sieves (80 mg). After 10 min stirring sodium triacetoxyborohydride (281 mg; 1.330 mmol) was added and the resulting mixture was stirred at ambient temperature for 12 hrs. Then reaction mixture was diluted with 6 mL methylene chloride and washed with saturated $NaHCO_3$ solution, brine, dried over $Na_2SO_4$ and concentrated. The residue in 5 mL acetonitrile was added $K_2CO_3$ (276 mg; 1.990 mmol) followed by the dropwise addition of propargyl bromide (237 mg; 1.990 mmol) and the resulting mixture was stirred at ambient temperature for 12 hrs. The reaction mixture was partitioned between methylene chloride and water. Aqueous layer was extracted with methylene chloride, and the combined organic layers were washed with brine, dried over $Na_2SO_4$ and concentrated under reduced pressure. The residue was purified by flash chromatography (hexanes/EtOAc=90/10-70/30) to afford the desired compound as colorless oil (200 mg; 87%). $^1$H NMR (400 MHz, $CDCl_3$): δ 5.40 (br s, 1H), 3.47-3.34 (m, 2H), 3.31-3.09 (m, 3H), 2.77-2.68 (m, 1H), 2.67-2.58 (m, 1H), 2.31-2.22 (m, 1H), 2.18-2.14 (m, 1H), 2.12-2.03 (m, 1H), 1.94-1.84 (m, 2H), 1.83-1.74 (m, 2H), 1.68-1.58 (m, 2H), 1.41 (s, 9H), 1.18 (s, 3H), 1.08 (d, *J*=7.02 Hz, 3H), 0.98 (s, 3H), 0.85 (d, *J*=9.86 Hz, 1H). $^{13}$C NMR (400 MHz, $CDCl_3$): δ 156.1, 81.5, 78.7, 72.0, 60.8, 48.1, 47.4, 41.7, 40.8, 40.3, 39.9, 39.1, 33.4, 28.5, 28.2, 28.0, 27.0, 23.4, 21.4. HRMS calcd for $C_{21}H_{37}N_2O_2$ $[M+H]^+$ 349.2855, found 349.2853. $\alpha_{589}^{23}$–45 (c 0.75, $CHCl_3$).

**N[1]-(Prop-2-yn-1-yl)-N[1]-((1R,2R,3R,5S)-2,6,6-trimethylbicyclo[3.1.1]heptan-3-yl)propane-1,3-diamine (27).** To compound **26** (186 mg; 0.533 mmol) in 5 mL methylene chloride was added 4M HCl in 1,4-dioxane (1.33 mL, 10 eq) and the resulting mixture was stirred at ambient temperature for 12 hrs. Reaction mixture was diluted with methylene chloride and water and pH of an aqueous layer was adjusted to 9 with aqueous ammonia. Aqueous layer was extracted with methylene chloride three times. Combined organic layers were dried over $Na_2SO_4$ and concentrated. The residue was dried in vacuo to afford the desired compound as yellow oil (132 mg, quant). Compound **27** was used in the next step without purification. $^1$H NMR (400 MHz, $CDCl_3$): δ 3.48-3.36 (m, 2H), 3.29-3.21 (m, 1H), 2.79-2.67 (m, 3H), 2.63-2.55 (m, 1H), 2.30-2.22 (m, 1H), 2.16-2.13 (m, 1H), 2.11-2.03 (m, 1H), 1.94-1.74 (m, 4H), 1.63-1.55 (m, 2H), 1.18 (s, 3H), 1.07 (d, *J*=7.00 Hz, 3H), 0.98 (s, 3H), 0.85 (d, *J*=9.85 Hz, 1H).

**2,3,4,5,6-Pentafluoro-N-(3-(prop-2-yn-1-yl((1R,2R,3R,5S)-2,6,6-trimethylbicyclo[3.1.1]heptan-3-yl)amino)propyl)benzamide (28).** To pentafluorobenzoic acid (68 mg; 0.320 mmol) in 3 mL methylene chloride was added 1-hydroxybenzotriazole (48 mg; 0.353 mmol), followed by the addition of 1-ethyl-3-(3-dimethylaminopropyl)carbodiimide hydrochloride (68 mg; 0.353 mmol). After 10 min of stirring, crude compound **27** (79 mg; 0.320 mmol) was added and the resulting mixture was stirred at ambient temperature for 12 hrs. Then reaction mixture was diluted with methylene chloride and water and pH of an aqueous layer was adjusted to 8 with $NaHCO_{3sat}$. Aqueous layer was extracted with methylene chloride three times. Combined organic layers were washed with brine, dried over $Na_2SO_4$ and concentrated. The residue was purified by flash chromatography (hexanes/EtOAc=90/10-70/30) to afford the desired compound as yellow oil (98 mg; 70%). $^1$H NMR (400 MHz, $CDCl_3$): δ 8.06 (br s, 1H), 3.86-3.76 (m, 1H), 3.45-3.25 (m, 4H), 2.88-2.79 (m, 1H), 2.75-2.67 (m, 1H), 2.31-2.23 (m, 1H),

2.07-1.98 (m, 2H), 1.94-1.67 (m, 6H), 0.95 (s, 3H), 0.91 (d, *J*=7.03 Hz, 3H), 0.78 (d, *J*=9.99 Hz, 1H). $^{13}$C NMR (400 MHz, $CDCl_3$): δ 156.8; 143.8 (dm, $J_{C-F}$=251.8 Hz), 141.9 (dm, $J_{C-F}$=256.5 Hz), 137.44 (dm, $J_{C-F}$=255.2 Hz), 112.5 (br t, $J_{C-F}$=20.9 Hz), 80.7; 72.0; 60.0; 48.5; 47.9; 41.5; 40.5; 40.4; 39.1; 33.2; 27.9; 27.3; 25.2; 23.3; 20.9. $^{19}$F NMR (400 MHz, $CDCl_3$): δ -140.60 (m, 2F), -152.20 (br t, *J*=20.56 Hz, 1F), -160.77 (m, 2F). HRMS calcd for $C_{23}H_{28}N_2OF_5$ $[M+H]^+$ 443.2122, found 443.2127. $\alpha_{589}^{23}$ 5 (c 1.62, $CHCl_3$).

**4-Azido-2,3,5,6-tetrafluoro-N-(3-(prop-2-yn-1-yl((1R,2R,3R,5S)-2,6,6-trimethylbicyclo[3.1.1]heptan-3-yl)amino)propyl)benzamide (9VP40).** To compound **28** (59 mg; 0.133 mmol) in 0.5 mL of dimethylformamide were added sodium azide (10.4 mg; 0.160 mmol) and tetrabutylammonium azide (3.8 mg; 0.013 mmol). Reaction flask was wrapped with tin foil and reaction mixture was stirred at ambient temperature for 12 hrs. Then reaction mixture was poured onto ice/water mixture and extracted with ethyl acetate. The organic layer was washed with water, brine, dried over $Na_2SO_4$ and concentrated. The residue was immediately purified by flash chromatography (hexanes/EtOAc=80/20-70/30) to afford the desired compound as clear oil (30 mg; 48%). The product was stored under nitrogen in air-tight container at -20°C. $^1$H NMR (400 MHz, $CDCl_3$): δ 7.95 (br s, 1H), 3.83-3.74 (m, 1H), 3.46-3.27 (m, 4H), 2.88-2.79 (m, 1H), 2.76-2.68 (m 1H), 2.31-2.23 (m, 1H), 2.08-1.98 (m, 2H), 1.95-1.68 (m, 6H), 1.18 (s, 3H), 0.98 (s, 3H), 0.93 (d, *J*=7.04 Hz, 3H), 0.78 (d, *J*=10.00 Hz, 1H). $^{19}$F NMR (400 MHz, $CDCl_3$): δ -140.79 (m, 2F), -150.91 (m, 2F). $^{13}$C NMR (400 MHz, $CDCl_3$): δ 157.2; 143.9 (doublet of multiplets, $J_{C-F}$=252.0 Hz), 140.4 (doublet of multiplets, $J_{C-F}$=251.7 Hz), 121.3 (br t, $J_{C-F}$=12.3 Hz), 112.5 (t, $J_{C-F}$=19.5 Hz), 80.7, 72.2, 60.2, 48.5, 47.9, 41.5, 40.6, 40.5, 40.1, 39.1, 33.2, 27.9, 27.5, 25.2, 23.3, 21.0. HRMS calcd for $C_{23}H_{28}N_5OF_4$ $[M+H]^+$ 466.2230, found 466.2237. $\alpha_{589}^{23}$ 4 (c 1.52, $CHCl_3$).

## 1.2 HPLC data for 1-12 and PRP

**Supplementary Figure 8.** HPLC chromatogram for 8VP70 (10VP28).

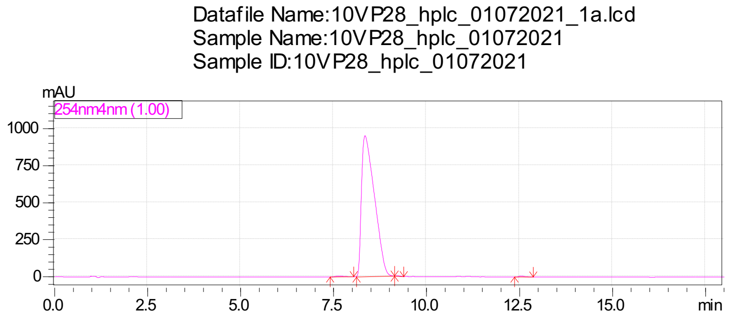


**Supplementary Table 2.** HPLC peak quantification for 8VP70 (10VP28).

| Peak# | Retention Time | Area | Height | Area% |
|---|---|---|---|---|
| 1 | 7.642 | 130734 | 7091 | 0.561 |
| 2 | 8.344 | 23057713 | 950378 | 98.956 |
| 3 | 9.237 | 28165 | 3323 | 0.121 |
| 4 | 12.541 | 84339 | 7551 | 0.362 |

**Supplementary Figure 9.** HPLC chromatogram for 8VP71.

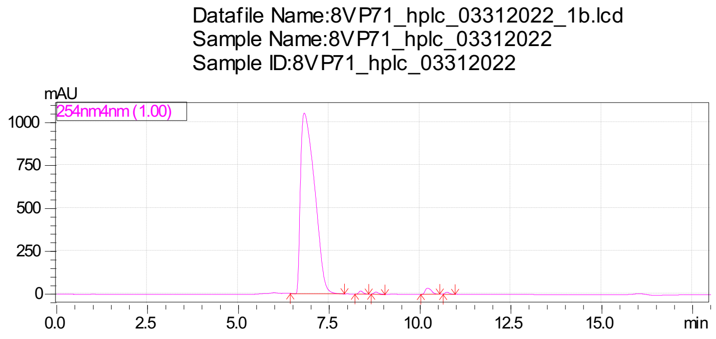


**Supplementary Table 3.** HPLC peak quantification for 8VP71.

| Peak# | Retention Time | Area | Height | Area% |
|---|---|---|---|---|
| 1 | 6.813 | 28381506 | 1054416 | 97.297 |
| 2 | 8.363 | 144240 | 18421 | 0.494 |
| 3 | 8.785 | 125437 | 13348 | 0.430 |
| 4 | 10.203 | 415124 | 35148 | 1.423 |
| 5 | 10.739 | 103522 | 12520 | 0.355 |
| Total | | 29169829 | 1133853 | 100.000 |

**Supplementary Figure 10.** HPLC chromatogram for 8VP83 (10VP76).

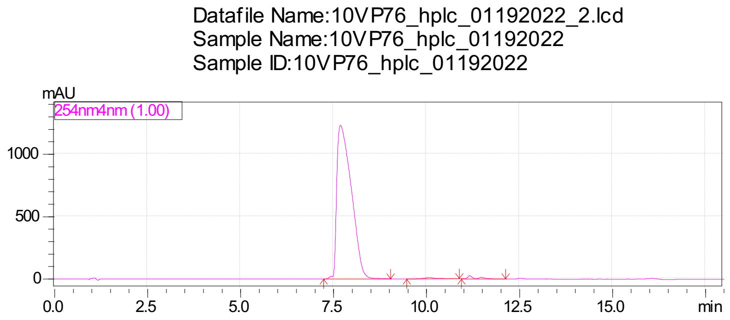


**Supplementary Table 4.** HPLC peak quantification for 8VP83 (10VP76).

| Peak# | Retention Time | Area | Height | Area% |
|---|---|---|---|---|
| 1 | 7.685 | 33616808 | 1233274 | 97.979 |
| 2 | 10.076 | 282802 | 12249 | 0.824 |
| 3 | 11.157 | 410672 | 25938 | 1.197 |
| Total | | 34310282 | 1271461 | 100.000 |

**Supplementary Figure 11.** HPLC chromatogram for 8VP101 (9VP21).

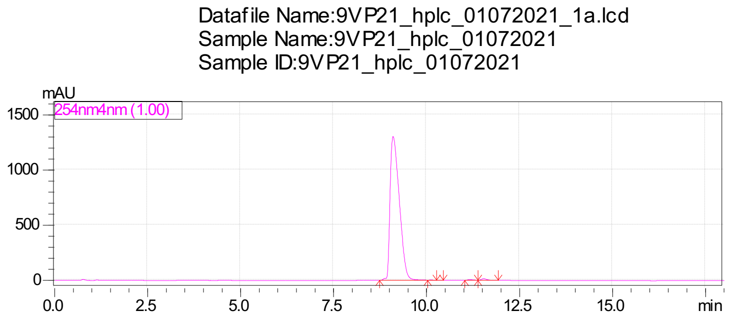


**Supplementary Table 5.** HPLC peak quantification for 8VP101 (9VP21).

| Peak# | Retention Time | Area | Height | Area% |
|---|---|---|---|---|
| 1 | 9.100 | 22673443 | 1306755 | 99.057 |
| 2 | 10.127 | 5580 | 787 | 0.024 |
| 3 | 11.161 | 64840 | 6549 | 0.283 |
| 4 | 11.535 | 145458 | 14347 | 0.635 |
| Total | | 22889321 | 1328438 | 100.000 |

**Supplementary Figure 12.** HPLC chromatogram for 8VP121-1 (9VP105).

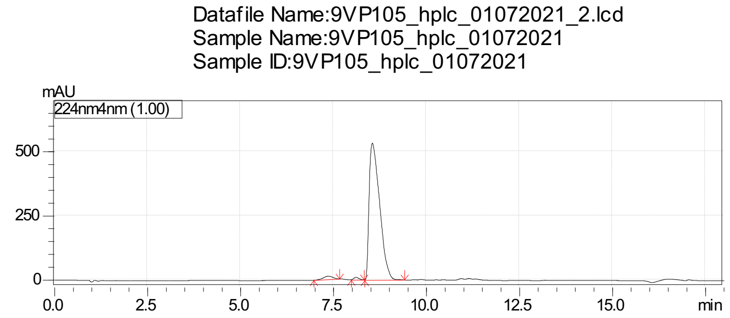


**Supplementary Table 6.** HPLC peak quantification for 8VP121-1 (9VP105).

| Peak# | Retention Time | Area | Height | Area% |
|---|---|---|---|---|
| 1 | 7.353 | 276808 | 14224 | 2.540 |

| | | | | |
|---|---|---|---|---|
| 2 | 8.102 | 107574 | 10913 | 0.987 |
| 3 | 8.541 | 10512720 | 535156 | 96.473 |
| Total | | 10897102 | 560294 | 100.000 |

**Supplementary Figure 13.** HPLC chromatogram for 8VP131.

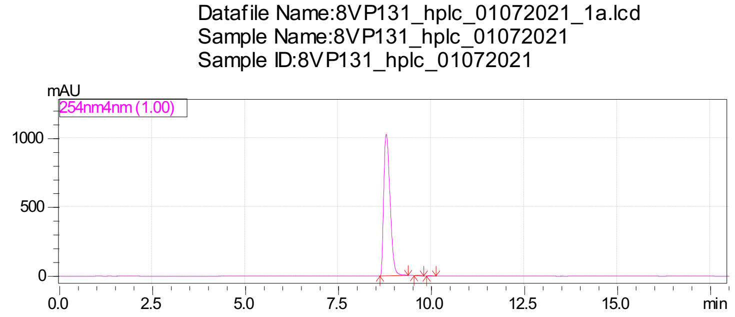


**Supplementary Table 7.** HPLC peak quantification for 8VP131.

| Peak# | Retention Time | Area | Height | Area% |
|---|---|---|---|---|
| 1 | 8.780 | 12148864 | 1028307 | 99.642 |
| 2 | 9.629 | 28340 | 3925 | 0.232 |
| 3 | 9.961 | 15254 | 1806 | 0.125 |
| Total | | 12192457 | 1034038 | 100.000 |

**Supplementary Figure 14.** HPLC chromatogram for 8VP192 (10VP30).

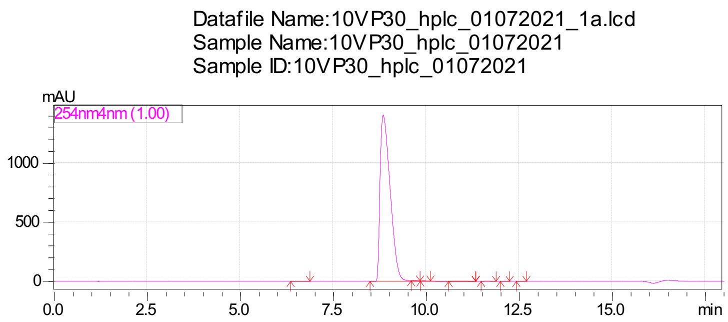


**Supplementary Table 8.** HPLC peak quantification for 8VP192 (10VP30).

| Peak# | Retention Time | Area | Height | Area% |
|---|---|---|---|---|
| 1 | 6.577 | 7724 | 494 | 0.029 |
| 2 | 8.834 | 26618607 | 1409093 | 99.616 |
| 3 | 9.692 | 23914 | 3399 | 0.089 |
| 4 | 9.933 | 14776 | 2071 | 0.055 |
| 5 | 10.861 | 35787 | 2009 | 0.134 |
| 6 | 11.632 | 14231 | 1476 | 0.053 |
| 7 | 12.122 | 1896 | 214 | 0.007 |
| 8 | 12.519 | 4409 | 539 | 0.017 |
| Total | | 26721345 | 1419294 | 100.000 |

**Supplementary Figure 15.** HPLC chromatogram for 9VP51.

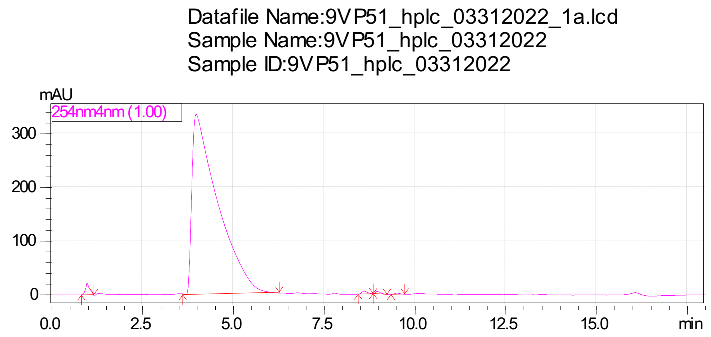


**Supplementary Table 9.** HPLC peak quantification for 9VP51.

| Peak# | Retention Time | Area | Height | Area% |
|---|---|---|---|---|
| 1 | 0.967 | 172312 | 21697 | 1.025 |
| 2 | 3.972 | 16525201 | 335498 | 98.280 |
| 3 | 8.599 | 56565 | 5177 | 0.336 |
| 4 | 8.961 | 41116 | 4252 | 0.245 |
| 5 | 9.504 | 19201 | 1963 | 0.114 |
| Total | | 16814395 | 368586 | 100.000 |

**Supplementary Figure 16.** HPLC chromatogram for 9VP108 (9VP196).

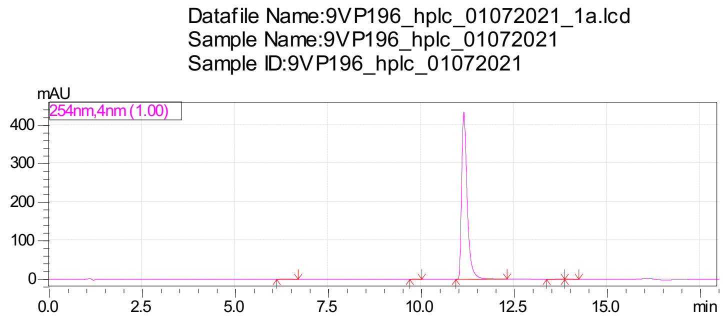


**Supplementary Table 10.** HPLC peak quantification for 9VP108 (9VP196).

| Peak# | Retention Time | Area | Height | Area% |
|---|---|---|---|---|
| 1 | 6.347 | 15543 | 965 | 0.338 |
| 2 | 9.810 | 8296 | 1005 | 0.180 |
| 3 | 11.135 | 4551078 | 434314 | 99.001 |
| 4 | 13.797 | 12212 | 778 | 0.266 |
| 5 | 13.849 | 9890 | 721 | 0.215 |
| Total | | 4597018 | 437782 | 100.000 |

**Supplementary Figure 17.** HPLC chromatogram for 9VP128-2 (10VP107).

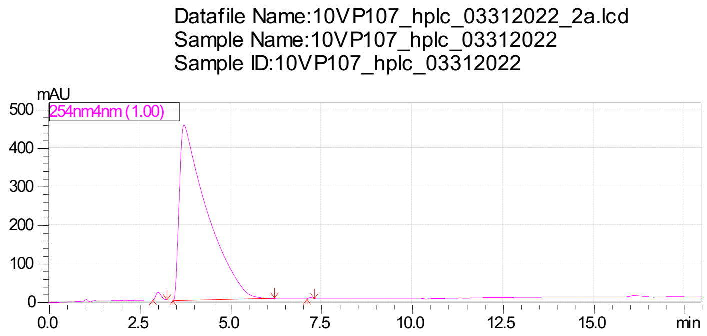


**Supplementary Table 11.** HPLC peak quantification for 9VP128-2 (10VP107).

| Peak# | Retention Time | Area | Height | Area% |
|---|---|---|---|---|
| 1 | 2.995 | 211343 | 19980 | 0.867 |
| 2 | 3.707 | 24136845 | 455441 | 99.004 |
| 3 | 7.189 | 31543 | 3732 | 0.129 |
| Total | | 24379730 | 479154 | 100.000 |

**Supplementary Figure 18.** HPLC chromatogram for (9VP173, 10VP24).

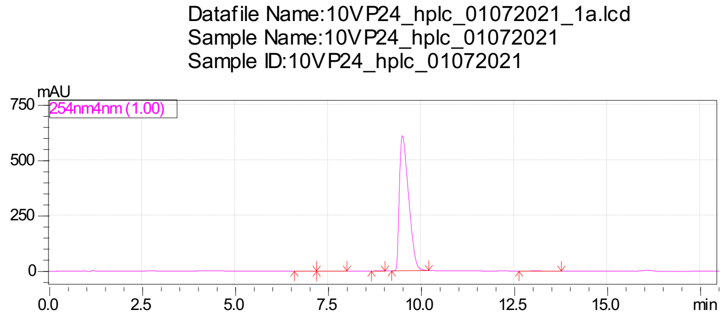


**Supplementary Table 12.** HPLC peak quantification for (9VP173, 10VP24).

| Peak# | Retention Time | Area | Height | Area% |
|---|---|---|---|---|
| 1 | 6.846 | 4404 | 220 | 0.042 |
| 2 | 7.418 | 5042 | 235 | 0.048 |
| 3 | 8.809 | 4719 | 418 | 0.045 |
| 4 | 9.483 | 10539390 | 610653 | 99.659 |
| 5 | 13.098 | 21927 | 636 | 0.207 |
| Total | | 10575482 | 612163 | 100.000 |

**Supplementary Figure 19.** HPLC chromatogram for 10VP91.

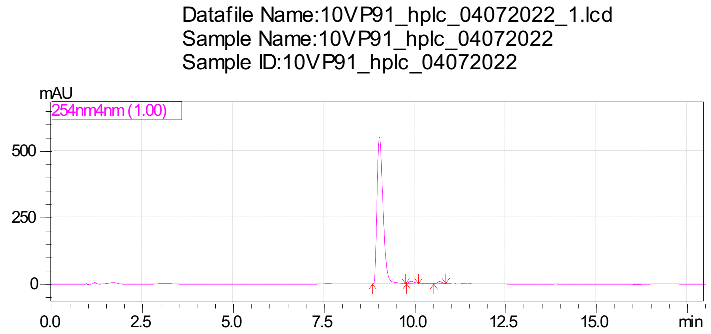

**Supplementary Table 13.** HPLC peak quantification for 10VP91.

| Peak# | Retention Time | Area | Height | Area% |
|---|---|---|---|---|
| 1 | 9.018 | 6407381 | 553392 | 97.446 |
| 2 | 9.894 | 100519 | 8984 | 1.529 |
| 3 | 10.669 | 67389 | 7961 | 1.025 |
| Total | | 6575290 | 570337 | 100.000 |

**Supplementary Figure 20.** HPLC chromatogram for 9VP40.

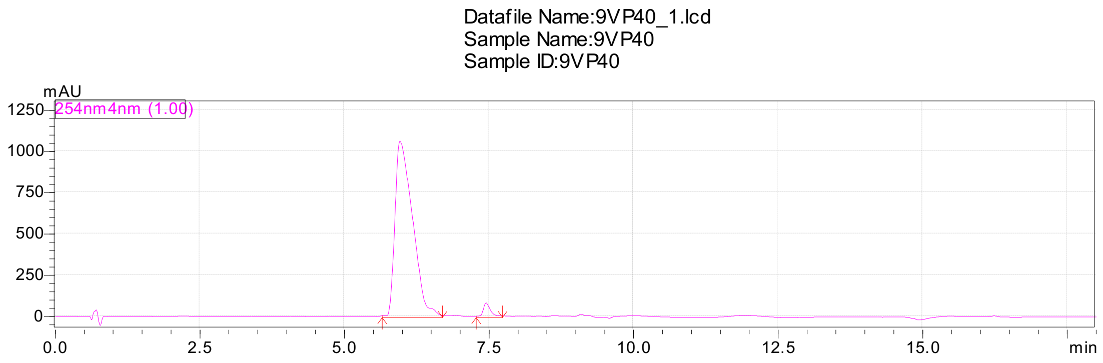


**Supplementary Table 14.** HPLC peak quantification for 9VP40.

| Peak# | Retention Time | Area | Height | Area% |
|---|---|---|---|---|
| 1 | 5.962 | 22805269 | 1067858 | 96.357 |
| 2 | 7.449 | 862239 | 87868 | 3.643 |
| Total | | 23667508 | 1155726 | 100.000 |

## 2 Supplementary Figures and Tables

**Supplementary Figure 21.** Inhibition of TGR in NTS visualized with TRFS-Green ($\lambda_{EX}$ = 438 nm, $\lambda_{EM}$ = 538 nm). Fluorescence quantification of treated NTS (AF @ 3 µM (blue), 1 µM (orange), 0.5 (gray) µM other compounds @ 30 µM (blue), 15 µM (orange), 5 µM (gray) and control (yellow, no compound addition) at the indicated times after TRFS-Green addition. Data are represented by two independent experiments as mean ± SD of biological replicates. Source data are provided as a Source Data file.

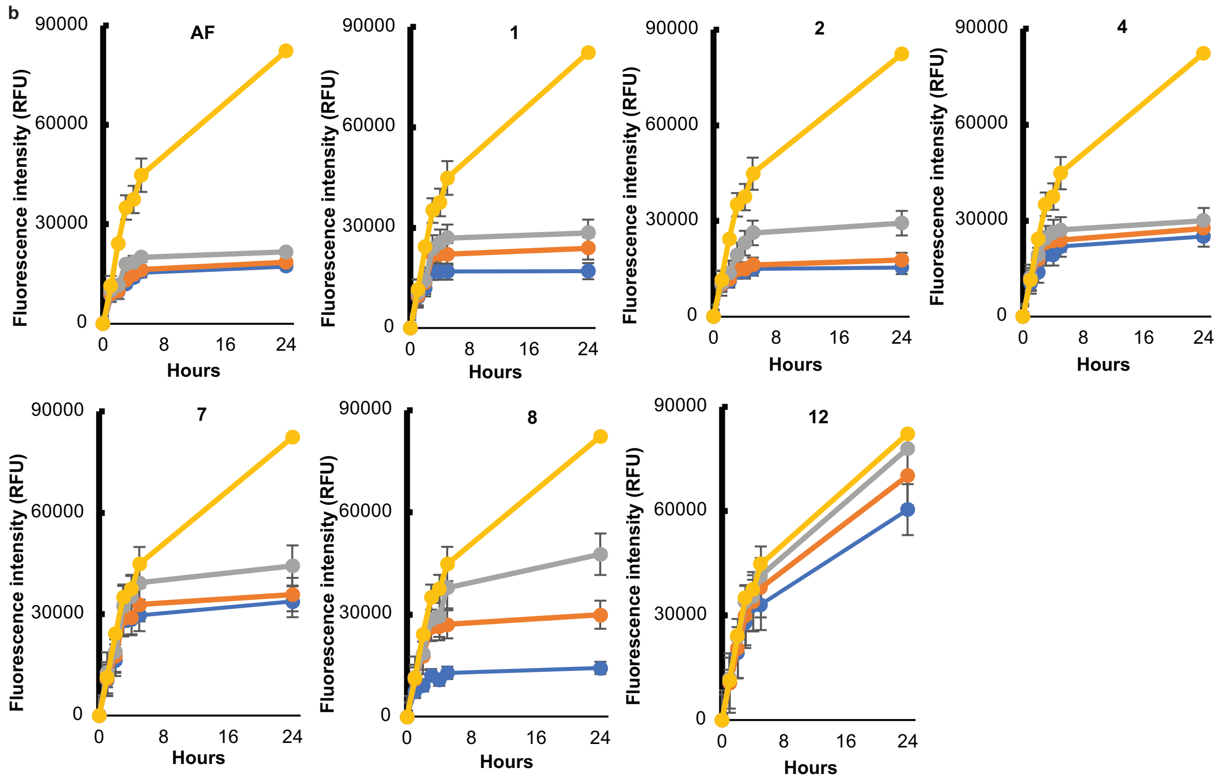

**Supplementary Figure 22.** LC MS chromatogram of covalent TGR inhibitor TRi-1. (a) UV profile of TRi-1, (b) LC-MS profile of TRi-1 where TIC@1 is a total ion current LC-MS chromatogram in positive mode, TIC@2 is a total ion current LC-MS chromatogram in negative mode, and 329.00@1 is single mass LC-MS chromatogram for m/z=329.00 corresponding to TRi-1, (c) Mass-spectra of TRi-1 as determined at retention time 9.15 min.

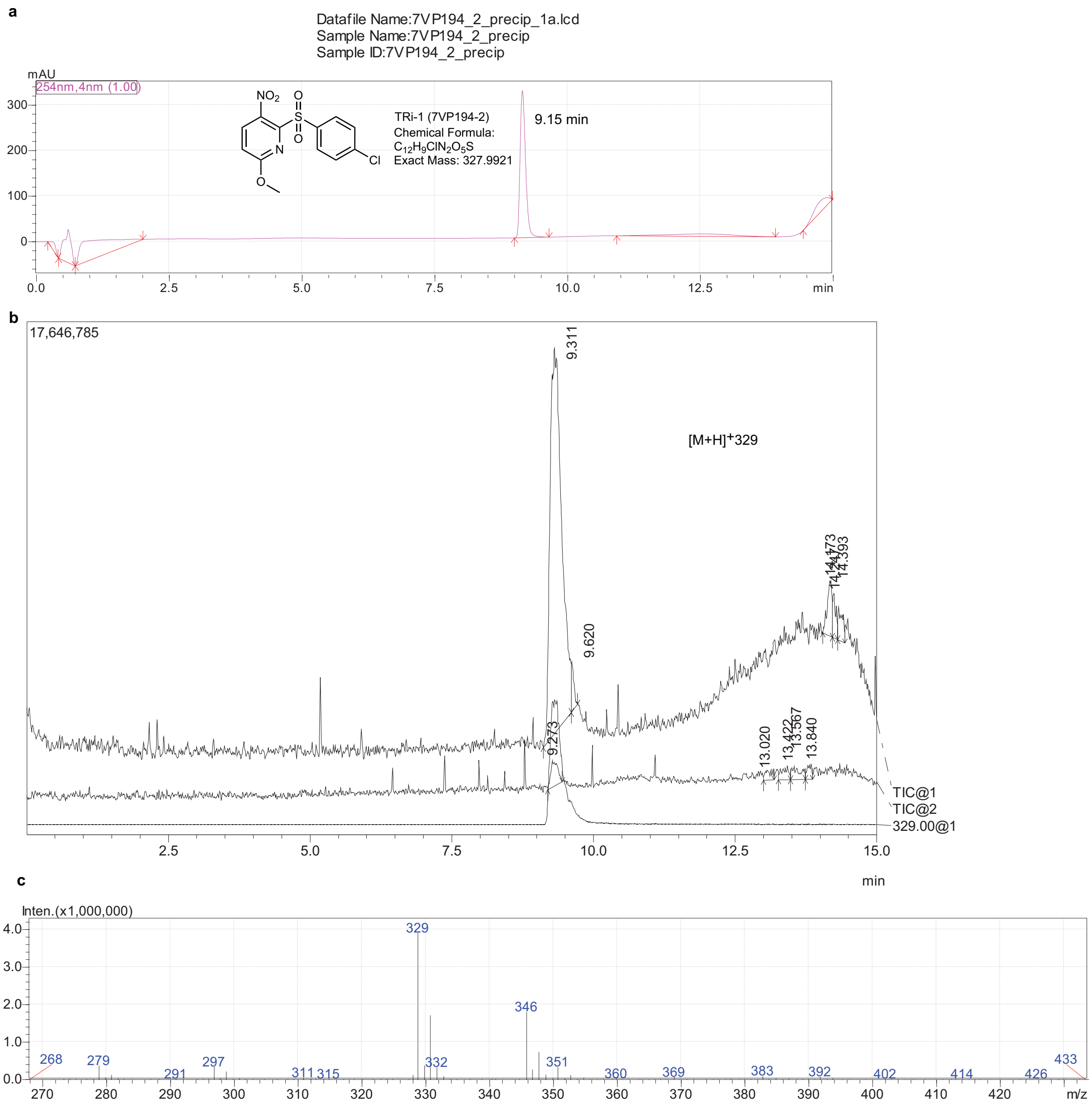

**Supplementary Figure 23.** LC MS chromatogram of a covalent adduct between TRi-1 and N-Boc protected methyl ester of L-selenocysteine. A single adduct between TRi-1 and N-Boc protected methyl ester of L-selenocysteine was detected by LCMS and TLC (not shown) analysis. (a) a UV profile of an adduct between TRi-1 and N-Boc protected methyl ester of L-selenocysteine. There is no visible peak for TRi-1 in the reaction mixture 9.15 min and, instead, a new peak is observed at 9.95 min retention time. (b) LC-MS profile of a TRi-1 and N-Boc protected methyl ester of L-selenocysteine adduct, its corresponding structure and the structure of two putative in-source generated derivatives, and the calculated isotopic peak distribution of these ions. TIC@1 is a total ion current LC-MS chromatogram in positive mode, TIC@2 is a total ion current LC-MS chromatogram in negative mode, 458.00@1 is a single mass LC-MS chromatogram corresponding to TRi-1, N-Boc protected methyl ester of L-selenocysteine, and sodium ion adduct $[M+Na]^+$, 380.00@1 and 336.00@1 are single mass LC-MS chromatograms corresponding to the likely generated in-source derivatives with *m/z* 335 *m/z* 379, (c) Isotopic peak distribution patterns of all the three signals - *m/z* $[M+Na]^+$ 458 and *m/z* $[M+H]^+$ 336 and 380 confirm presence of selenium atom in their structures.

**a**

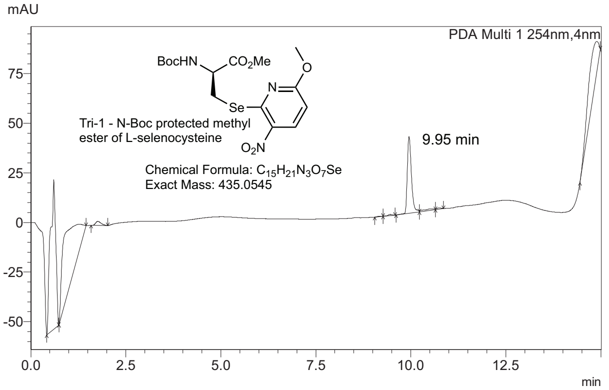


**b**

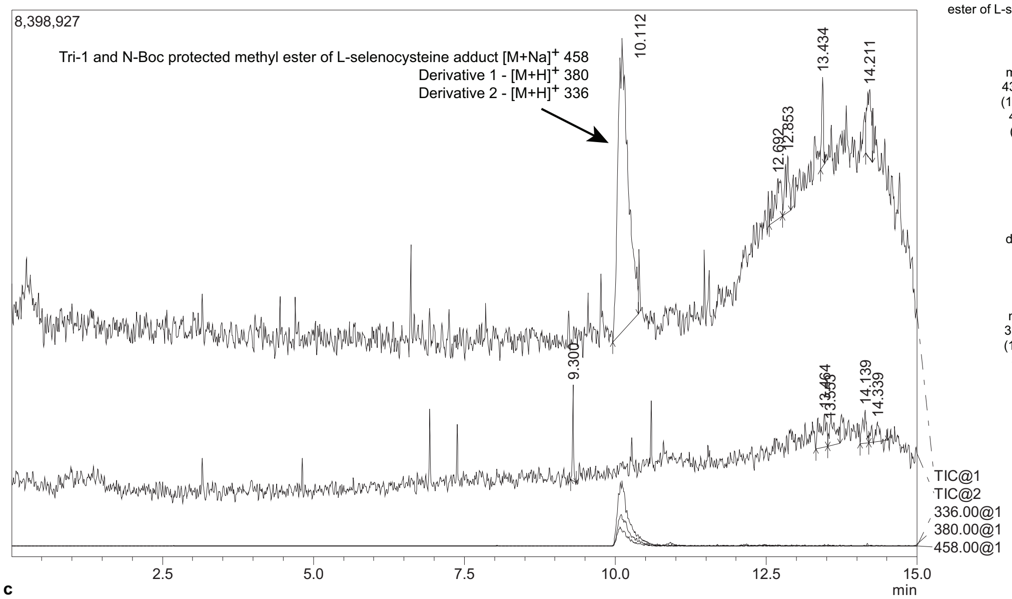


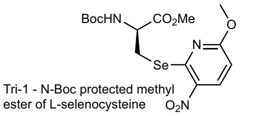


Chemical Formula: $C_{15}H_{21}N_3O_7Se$
Exact Mass: 435.05
m/z: 435.05 (100.0%), 433.06 (50.7%), 431.06 (18.6%), 432.06 (18.4%), 437.05 (17.9%), 436.06 (16.9%), 434.06 (8.4%), 438.06 (3.3%), 437.06 (2.8%), 429.06 (1.7%), 435.06 (1.4%), 436.05 (1.1%)

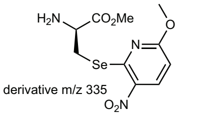


Chemical Formula: $C_{10}H_{13}N_3O_5Se$
Exact Mass: 335.00
m/z: 335.00 (100.0%), 333.00 (47.7%), 331.00 (18.5%), 337.00 (17.8%), 332.01 (17.3%), 336.01 (11.2%), 334.01 (5.6%), 338.01 (2.1%), 333.01 (2.0%), 329.01 (1.7%), 337.01 (1.6%), 336.00 (1.1%)

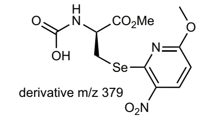


Chemical Formula: $C_{11}H_{13}N_3O_7Se$
Exact Mass: 378.99
m/z: 378.99 (100.0%), 376.99 (47.7%), 374.99 (18.5%), 380.99 (17.8%), 376.00 (17.5%), 380.00 (12.4%), 378.00 (6.2%), 382.00 (2.4%), 377.00 (2.3%), 381.00 (2.1%), 373.00 (1.7%), 379.99 (1.1%), 379.00 (1.0%)

**c**

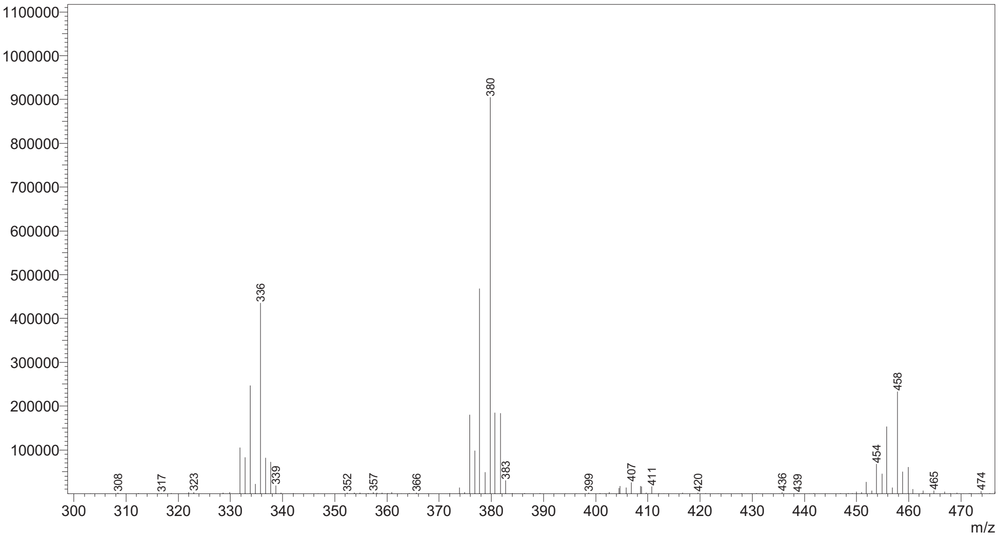

**Supplementary Figure 24.** Western Blot of recombinant TGR and recombinant SmHDAC8 prepared with 5 or 50 µM **PRP** (9VP40) and 100 µM (+) or 0 µM (-) NADPH followed by photocrosslinking and CuAAC click biotinylation as described previously.[17] 800 nm bands show biotin-streptavidin labeling of IRDye® 800CW streptavidin that recognize the biotinylated PRP. 700 nm bands show total protein staining for normalization. (n=3 independent experiments for labeling of TGR, n=1 independent experiments for labeling of SmHDAC8).

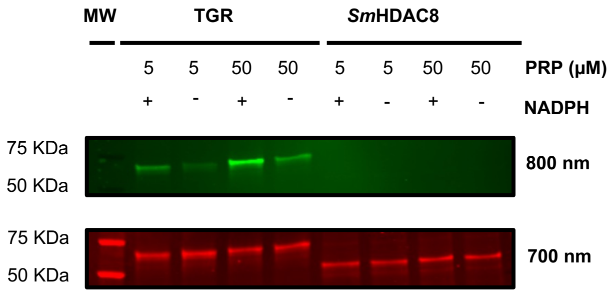

**Supplementary Figure 25.** Overall and local resolution of the TGR-**9** (9VP128-2) cryo-EM map. a) a negative staining image of a sample of the TGR-**9** (9VP128-2) complex. b) Micrograph of a sample of the TGR-**9** (9VP128-2) complex collected on a Glacios cryo-TEM (ThermoFisher) operating at 200 kV; scalebar = 40 nm. c) 2D class averages d) The plot shows the Fourier shell correlation (FSC) curve of the final calculated cryo-EM map. The reported resolution is based on the FSC = 0.143 criterion. e) representative secondary structural elements of TGR are shown to illustrate the quality of the cryo-EM maps and of the refined atomic model. f) Angular distribution plot of all TGR particles that contributed to the final map. The map and the angular distribution plot have the same orientation. The height and colour (from blue to red) of the cylinder bars are proportional to the number of particles in those views. g) Local resolution variations in the cryo-EM map. The resolution ranges from 3.5 to 4.9 Å, as calculated by the Local resolution program of the Phenix suite.

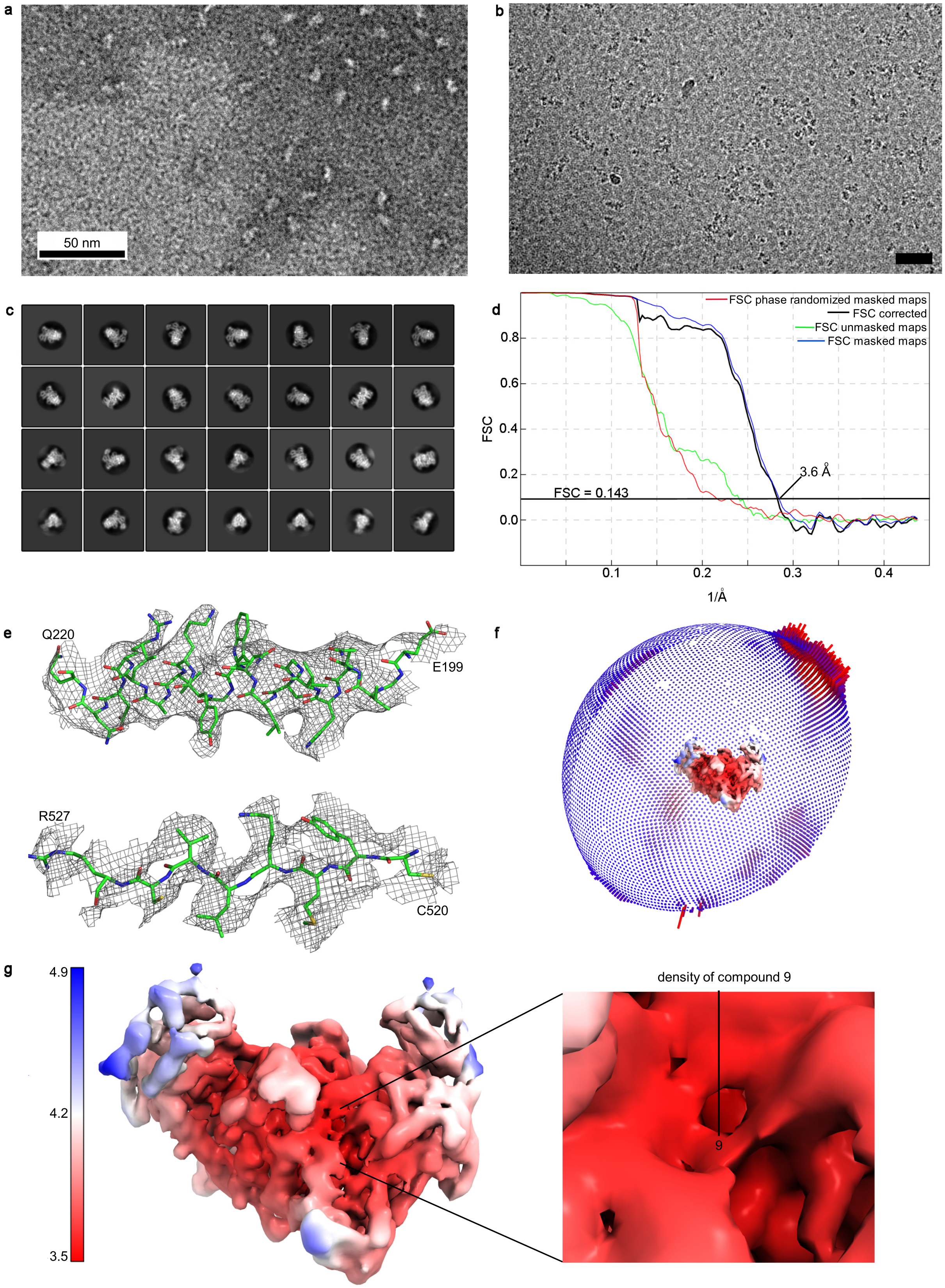
a
50 nm
b
c
d
FSC phase randomized masked maps
FSC corrected
FSC unmasked maps
FSC masked maps
FSC
1/Å
3.6 Å
FSC = 0.143
e
Q220
E199
R527
C520
f
g
4.9
4.2
3.5
density of compound 9
9

**Supplementary Figure 26.** The cryo-EM map of the TGR-9 complex. Magnification of the cryo-EM map of the TGR-**9** (9VP-128-2) complex at the doorstop pocket showing the pinane ring of the compound in the subpocket C and the main surrounding residues.

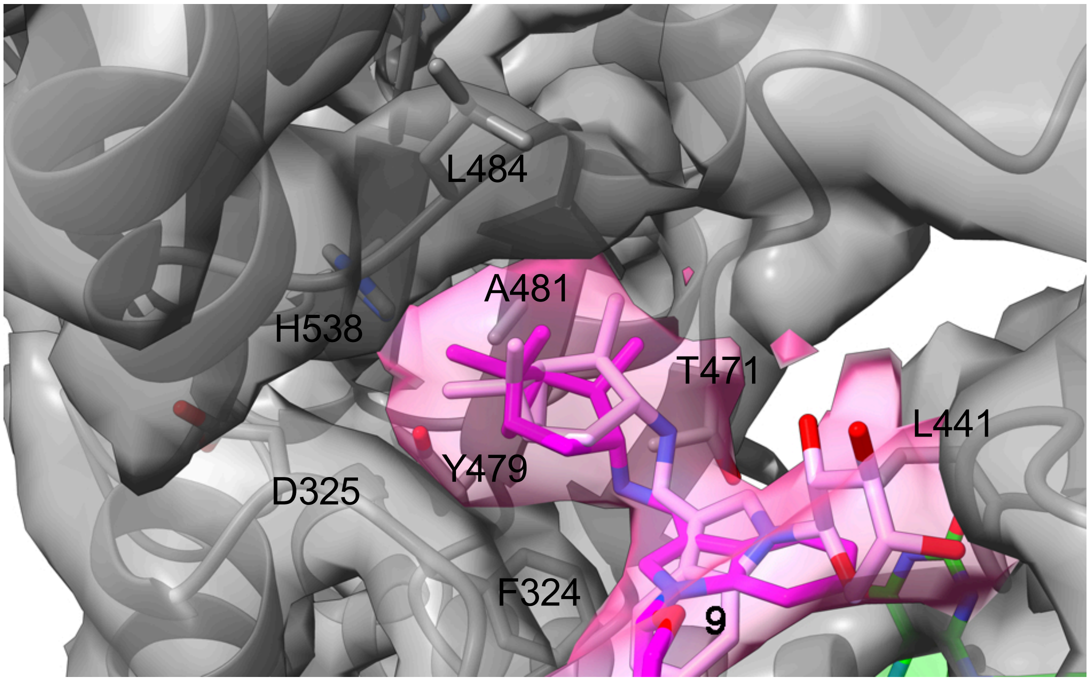

**Supplementary Figure 27.** Sequence alignment of pyridine nucleotide-disulfide oxidoreductases of interest in this study. The PDB IDs of each protein are indicated in parenthesis. The side-chain residues contributing to the doorstop pocket in SmTGR as found by the Cast-p calculation are highlighted in yellow.[18, 19] When a residue is not conserved with respect to SmTGR, the green color is used. Side chain residues within 5 Å which are involved in the recognition of compound **9** are indicated by a "$" symbol. Overall percentages of identity with respect to SmTGR: SjTGR (91%); hTrxR1 (61%), BmTrxR-d (51%), PfTrxR (47%), hGR (37%). Percentages of identity with respect to the residues forming the doorstop pocket in TGR: SjTGR (100%), hTrxR1 (74%), BmTrxR-d (76%), PfTrxR (72%), hGR (60%).

SmTGR: 2V6O https://www.ncbi.nlm.nih.gov/Structure/pdb/2V6O

SjTGR: 4LA1_A https://www.ncbi.nlm.nih.gov/protein/4LA1_A

BmTrxR-d: 7P0X_A https://www.ncbi.nlm.nih.gov/protein/7P0X_A

hTrxR1: NP_877393.1 https://www.ncbi.nlm.nih.gov/protein/33519426

PfTrxR: Q25861.1 https://www.ncbi.nlm.nih.gov/protein/Q25861.1

hGR: 3DK8_A https://www.ncbi.nlm.nih.gov/protein/3DK8_A

```
SmTGR(2v6o)         MPP------ADGTSQWLRKTVDSAAVILFSKTTCPYCKKVKDVLAEAKIKHATIELDQLSNGSAIQKCLASFSKIETVPQ  74
SjTGR(4ALA1)        MPP------IDGTSQWLQRTIESAAVIVFSKTTCPFCKKLKDVLAEAKIKHATIELDQLSNGSVIQKALSNFSKIETVPQ  74
hTrxR1(2cfy)        M-----------------------------------------------------------NGP------------EDLPK  9
BmTrxR-d(7p0x)      MSPIPNRVSSGSLADAVFKSACEERILLAYADYNPDMTKVVNLFS--KYNETVNTVR-VSNDAV--KDILEIVGWPSMPL  75
PfTrxR(4b1b)        MCKDKN--------------------------------------EKKNYEHVNANEK---NGYL----------------  23
hGR(3dk8)           --------------------------------------------------------------------------------

SmTGR               MFVRGKFIGDSQTVLKYYSNDELAGIVNE--SKYDYDLIVIGGGSGGLAAGKEAAKYGAKTAVLDYVEPTPIGTTWGLGG  152
SjTGR               MFVRGKFIGDSKAVLNYHNNNQLQAIVNE--NKYDYDLIIIGGGSGGLAAGKEAAKYGAKTAVLDYVEPTPMGTTWGLGG  152
HsTrxR1             --------------------------------SYDYDLIIIGGGSGGLAAAKEAAQYGKKVMVLDFVTPTPLGTRWGLGG  57
BmTrxR              IFVKGNCCGGFKELYQLEE----SGFLNEWLKEHEYDLAIVGGGSGGLAAAKEAVRLGKKVVCLDFVKPSAMGTTWGLGG  151
PfTrxR              ---------------ASEKNELTKNKVEE--HTYDYDYVVIGGGPGGMASAKEAAAHGARVLLFDYVKPSSQGTKWGIGG  86
hGR                 ---------------ACRQEPQPQGPPPAAGAVASYDYLVIGGGSGGLASARRAAELGARAAVVESHK---------LGG  56

SmTGR               TCVNVGCIPKKLMHQAGLLSHALE-DAEHFGWSLDRSKISHNWSTMVEGVQSHIGSLNWGYKVALRDNQVTYLNAKGRLI  231
SjTGR               TCVNVGCIPKKLMHQAGLLSHSLE-DAQHFGWSLDKSKISHDWSTMVEGVQSHIGSLNWGYKVSLRDNAVTYLNARGMLL  231
hTrxR1              TCVNVGCIPKKLMHQAALLGQALQ-DSRNYGWKVEET-VKHDWDRMIEAVQNHIGSLNWGYRVALREKKVVYENAYGQFI  135
BmTrxR              TCVNVGCIPKKLMHQAALLGEYIE-DAKKFGWEIPEGAIKLNWHQLKNAVQNHIASLNWGYRVQLKEKSVTYMNSYATFT  230
PfTrxR              TCVNVGCVPKKLMHYAGHMGSIFKLDSKAYGWKFDN--LKHDWKKLVTTVQSHIRSLNFSYMTGLRSSKVKYINGLAKLK  164
hGR                 TCVNVGCVPKKVMWNTAVHSEFMH-DHADYGFPSCEG--KFNWRVIKEKRDAYVSRLNAIYQNNLTKSHIEIIRGHAAFT  133

SmTGR               SPHEVQ--ITDKNQKVSTITGNKIILATGERPKYP--E-IPGAVEYGITSDDLFSLPYFPGKTLVIGASYVALECAGFLA  306
SjTGR               SPHEVQ--ITEKNKKVSTITGNKIILATGERPKYP--E-IPGAIEYGITSDDLFSLPYFPGKTLVVGASYVALECAGFLA  306
hTrxR1              GPHRIK--ATNNKGKEKIYSAERFLIATGERPRYL--G-IPGDKEYCISSDDLFSLPYCPGKTLVVGASYVALECAGFLA  210
BmTrxR              GSHELS--VKNKKGKVEKVTADRFLIAVGLRPRFP--D-VPGALECCISSDDLFSLPYNPGKTLCVGASYVSLECAGFLK  305
PfTrxR              DKNTVSYYLKGDLSKEETVTGKYILIATGCRPHIP--DDVEGAKELSITSDDIFSLKKDPGKTLVVGASYVALECSGFLN  242
hGR                 SDPKPTIEVSGKK-----YTAPHILIATGGMPSTPHESQIPGA-SLGITSDGFFQLEELPGRSVIVGAGYIAVEMAGILS  207
                                    $$$
SmTGR               SLGGDVTVMVRSI-LLRGFDQQMAEKVGDYMENHGVKFAKLCVPDEIKQ----LKVVDTENNKPGLLLVKGHYT-DG--K  378
SjTGR               SLGGDVTVMVRSI-LLRGFDQQMAEKVGDYMENHGVKFAKLCVPDEITQ----LKPVDTENNKPGLLLVKGHYT-DG--K  378
hTrxR1              GIGLDVTVMVRSI-LLRGFDQDMANKIGEHMEEHGIKFIRQFVPIKVEQ----I-----EAGTPGRLRVVAQSTNSE--E  278
BmTrxR              GIGNDVTVMVRSV-LLRGFDQDMAERIKKHMTERGVKFV-QCVPIKYER----LK--KPTDSEPGMIRVHTMQEDEDGTK  377
PfTrxR              SLGYDVTVAVRSI-VLRGFDQQCAVKVKLYMEEQGVMFKNGILPKKLTK----MDD-----------KILVEFSDKT---  303
hGR                 ALGSKTSLMIRHDKVLRSFDSMISTNCTEELENAGVEVLKFSQVKEVKKTLSGLEV-SMVTAVPGRLPVMTMIP------  280
                                                                                   $ $$
SmTGR               KFEEEFETVIFAVGREPQLSKVLCETVGVKL-DKNGRVVCTDDEQTTVSNVYAIGDINAGKPQLTPVAIQAGRYLARRLF  457
SjTGR               KFEEEFETVIFAVGREPQLSKLNCEAVGVKL-DKNGRVVCSDDEQTTVSNIYAIGDINAGKPQLTPVAIHAGRYLARRLF  457
hTrxR1              IIEGEYNTVMLAIGRDACTRKIGLETVGVKINEKTGKIPVTDEEQTNVPYIYAIGDILEDKVELTPVAIQAGRLLAQRLY  358
BmTrxR              EVTEDFNTVLMAIGRDAMTDDLGLDVVGVNR-AKSGKIIGRREQSVSCPYVYAIGDVLYGSPELTPVAIQAGKVLMRRLF  456
PfTrxR              --SELYDTVLYAIGRKGDIDGLNLESLNMNVNKSNNKIIADHLSCTNIPSIFAVGDVAENVPELAPVAIKAGEILARRLF  381
hGR                 ----DVDCLLWAIGRVPNTKDLSLNKLGIQT-DDKGHIIVDEFQNTNVKGIYAVGDVC-GKALLTPVAIAAGRKLAHRLF  354

                                $ $         $  $
SmTGR               A-GATELTDYSNVATTVFTPLEYGACGLSEEDAIEKYGDKDIEVYHSNFKPLEWTVAHRED-------------NVCYM  522
SjTGR               A-GATELTDYSNVATTVFTPLEYGACGLSEEDAIEKYGDNDIEVYHSHFKPLEWTVAHRED-------------NVCYM  522
hTrxR1              A-GSTVKCDYENVPTTVFTPLEYGACGLSEEKAVEKFGEENIEVYHSYFWPLEWTIPSRDN-------------NKCYA  423
BmTrxR              T-GSSELTEYDKIPTTVFTPLEYGSCGLSEYSAIQKYGKENINVYHNVFIPLEYAVTERKE-------------KTHCYC  522
PfTrxR              K-DSDEIMDYSYIPTSIYTPIEYGACGYSEEKAYELYGKSNVEVFLQEFNNLEISAVHRQKHIRAQKDEYDLDVSSTCLA  460
hGR                 EYKEDSKLDYNNIPTVVFSHPPIGTVGLTEDEAIHKYGIENVKTYSTSFTPMYHAVTKRKT--------------KCVM  419

SmTGR               KLVCRKSDNMRVLGLHVLGPNAGEITQGYAVAIKMGATKADFDRTIGIHPTCSETFTTLHVTKKSGVSPIV-SGCUG---  598
SjTGR               KLVCRISDNMRVLGLHVLGPNAGEITQGYAVAIKMGATKEDFDRTIGIHPTCSETFTTLHVTKRSGGSAAV-TGCUG---  598
hTrxR1              KIICNTKDNERVVGFHVLGPNAGEVTQGFAAALKCGLTKKQLDSTIGIHPVCAEVFTTLSVTKRSGASILQ-AGCUG---  499
BmTrxR              KLICLKNEQDLILGFHILTPNAGEITQGFAIALKFDAKKADFDRLIGIHPTVAENFTTLTLVKEDGQTLKA-TGCUG---  598
PfTrxR              KLVCLKNEDNRVIGFHYVGPNAGEVTQGMALALRLKVKKKDFDNCIGIHPTDAESFMNLFVTISSGLSYAAKGGCGGGKCG 541
hGR                 KMVCA-NKEEKVVGIHMQGLGCDEMLQGFAVAVKMGATKADFDNTVAIHPTSSEELVTLR--------------------  478
```

**Supplementary Table 15.** Steady state parameters for the slow inhibitor **3** (8VP83) indicate its uncompetitive behavior. The reaction rate was determined at different concentrations of NADPH and **3** (8VP83) after 6 hr. preincubations. Source data are provided as a Source Data file.

| **3** (8VP83) (μM) | $K_m$ (μM) | $V_{max}$ ($\Delta A_{412}$/min) |
|---|---|---|
| 0 | 8.0 ± 1.0 | 0.015 ± 0.0005 |
| 35 | 5.3 ± 0.5 | 0.011 ± 0.0002 |
| 50 | 5.0 ± 0.4 | 0.0096 ± 0.0002 |
| 70 | 5.0 ± 0.8 | 0.0086 ± 0.0003 |

**Supplementary Table 16.** Steady state parameters for fast inhibitors **7** (9VP108), **8** (9VP173), and **9** (9VP128-2). Both the $K_m$ and $V_{max}$ are decreased by **7** (9VP108) and **8** (9VP173) indicating uncompetitive behavior. For **9** (9VP128-2), the $K_m$ remains constant while the $V_{max}$ decreases indicating noncompetitive inhibition. Data are represented by three independent experiments as mean ± SD. Source data are provided as a Source Data file.

| **7** (9VP108) (μM) | $K_m$ (μM) | $V_{max}$ ($\Delta A_{412}$/min) |
|---|---|---|
| 0 | 13.0 ± 3.5 | 0.039 ± 0.0031 |
| 3 | 11.7 ± 3.3 | 0.037 ± 0.0031 |
| 10 | 7.5 ± 0.8 | 0.023 ± 0.0006 |
| 30 | 2.3 ± 1.0 | 0.005 ± 0.0004 |
| **8** (9VP173) (μM) | $K_m$ (μM) | $V_{max}$ ($\Delta A_{412}$/min) |

| | | |
|---|---|---|
| 0 | 13.9 ± 2.5 | 0.057 ± 0.0023 |
| 0.6 | 11.3 ± 1.8 | 0.05 ± 0.0017 |
| 10 | 1.8 ± 1.9 | 0.0091 ± 0.0007 |
| 30 | 1.3 ± 0.26 | 0.0067 ± 7E-5 |
| **9** (9VP128-2) (μM) | $K_m$ (μM) | $V_{max}$ ($\Delta A_{412}$/min) |
| 0 | 18.7 ± 2.9 | 0.017 ± 0.00083 |
| 10 | 16.2 ± 0.51 | 0.012 ± 0.00012 |
| 30 | 22.6 ± 5.1 | 0.0056 ± 0.00043 |
| 60 | 22.5 ± 4.9 | 0.0033 ± 0.00024 |

**Supplementary Table 17.** Cryo-EM data collection, refinement, and validation statistics.

| TGR in complex with compound **9 (9VP128-2)**<br>(EMDB-15084; https://www.ebi.ac.uk/emdb/EMD-15084)<br>(PDB 8A1R; https://www.rcsb.org/structure/8A1R) | |
|---|---|
| Data collection and processing | |
| Magnification | 36000 |
| Voltage (kV) | 200 |
| Electron exposure ($e^-/Å^2$) | 50 |
| Defocus range (μm) | -2.6, -1.8 |
| Pixel size (Å) | 1.145 |
| Symmetry imposed | C2 |
| Initial particle images (no.) | 1479588 |
| Final particle images (no.) | 173572 |
| Map resolution (Å)<br>FSC threshold | 3.6<br>0.143 |
| Map resolution range (Å) | 3.5-4.9 |
| Refinement | |
| Initial model used (PDB code) | 2V6O |
| Model resolution (Å)<br>FSC threshold | 3.2(3.9)<br>0.143(0.5) |
| Model composition<br>Non-hydrogen atoms<br>Protein residues<br>Ligands | <br>9286<br>1186<br>4 |
| B factors ($Å^2$)<br>Protein<br>Ligand | <br>123.1<br>102.0 |
| R.m.s. deviations<br>Bond lengths (Å)<br>Bond angles (°) | <br>0.004<br>1.0 |
| Validation<br>MolProbity score<br>Clashscore<br>Poor rotamers (%) | <br>1.94<br>11.56<br>0.0 |
| Ramachandran plot<br>Favored (%)<br>Allowed (%)<br>Disallowed (%) | <br>94.75<br>5.25<br>0.0 |

**Supplementary Table 18.** Plasma concentration of **1** (8VP70) and **2** (8VP101) after intraperitoneal administration in mice. Data are presented as mean values ± standard deviation, values are the average of n=3 blood collections at each time point. Source data are provided as a Source Data file.

| Compounds | Time (min) | | | | Units |
|---|---|---|---|---|---|
| | 0 | 30 | 60 | 120 | |
| **1** (8VP70) | 0 | 1513 ± 17 | 1209 ± 117 | 865 ± 102 | ng/mL |
| **1** (8VP70) | 0 | 4.107 ± 0.047 | 3.280 ± 0.318 | 2.348 ± 0.277 | µM |
| **2** (8VP101) | 0 | 1692 ± 216 | 1524 ± 158 | 1079 ± 202 | ng/mL |
| **2** (8VP101) | 0 | 4.805 ± 0.612 | 3.912 ± 0.674 | 3.060 ± 0.578 | µM |

**Supplementary Table 19.** Plasma concentration of **2** (8VP101) after oral gavage in mice. Data are presented as mean values ± standard deviation, values are the average of n=2 blood collections at each time point. Source data are provided as a Source Data file.

| Compounds | Time (min) | | | | Units |
|---|---|---|---|---|---|
| | 0 | 30 | 60 | 120 | |
| **2** (8VP101) | 0 | 252 ± 5 | 272 ± 2 | 140 ± 6 | ng/mL |
| **2** (8VP101) | 0 | 714 ± 14 | 771 ± 5 | 396 ± 18 | nM |

**Supplementary Table 20.** Inhibition of TrxRs from *Brugia malayi* and *Plasmodium falciparum*. Compounds were screened for inhibitory activity against *B. malayi* (BmTrxR) and *P. falciparum* (PfTrxR) TrxRs. $IC_{50}$s in μM. [a] - % inhibition at 50 μM, the highest concentration tested. [b] - no inhibition at 50 μM. Data are represented by three independent experiments as mean ± SD. Source data are provided as a Source Data file.

| Compound | $IC_{50}$ (μM) | |
|---|---|---|
| | BmTrxR | PfTrxR |
| **1 (8VP70)** | 37 ± 3.04 | n.i.[b] |
| **2 (8VP101)** | 13%[a] | n.i.[b] |
| **3 (8VP83)** | n.i.[b] | 12%[a] |
| **4 (8VP121-1)** | n.i.[b] | 18%[a] |
| **5 (8VP131)** | 9%[a] | n.i.[b] |
| **7 (9VP108)** | 11%[a] | n.i.[b] |
| **8 (9VP173)** | 67.7%[a] | n.i.[b] |
| **9 (9VP128-2)** | 13.7 ± 1.03 | 16%[a] |
| **10 (10VP91)** | 2.5 ± 1.04 | 32.9 ± 4.46 |

# 3 Supplementary References


1. Molecular Operating Environment (MOE), 2019.01; Chemical Computing Group LLC, 1010 Sherbooke St. West, Suite #910, Montreal, QC, Canada, H3A 2R7, 2019 ) (2019).

2. Lipinski CA, Lombardo F, Dominy BW, Feeney PJ. Experimental and computational approaches to estimate solubility and permeability in drug discovery and development settings. *Adv. Drug Del. Rev.* **23**, 3-25 (1997).

3. Goto Y, Yamazaki M, Hamana M. Studies on Azole Compounds .3. Reactions of Oxazole N-Oxides with Phosphoryl Chloride and Acetic Anhydride. *Chem. Pharm. Bull. (Tokyo)* **19**, 2050-2057 (1971).

4. Lee GT, Jiang XL, Vedananda TR, Prasad K, Repic O. A highly regioselective preparation of 4-chloromethyl-5-methyl-2-aryl-1,3-oxazoles. *Adv. Synth. Catal.* **346**, 1461-1464 (2004).

5. He S*, et al.* Development of an Aryloxazole Class of Hepatitis C Virus Inhibitors Targeting the Entry Stage of the Viral Replication Cycle. *J. Med. Chem.* **60**, 6364-6383 (2017).

6. Goerlitzer J*, et al.* Diarylcycloalkyl derivatives, process for their preparation and their use as pharmaceuticals. U.S.A. Patent US7160911B2 (2007).

7. Tanaka A*, et al.* Inhibitors of acyl-CoA : cholesterol O-acyltransferase. 2. Identification and structure-activity relationships of a novel series of N-alkyl-N-(heteroaryl-substituted benzyl)-N '-arylureas. *J. Med. Chem.* **41**, 2390-2410 (1998).

8. Giambasu GM*, et al.* IL4I1 Inhibitors and methods of use. EPO Patent WO2022US26626 (2021).

9. Dow RL, Hulin B, Clark DA. Oxazolidinedione hypoglycemic agents. U.S.A. Patent US5498621A (1996).

10. Meguro K, Tawada H, Sugiyama Y, Fujita T, Kawamatsu Y. Studies on antidiabetic agents. VII. Synthesis and hypoglycemic activity of 4-oxazoleacetic acid derivatives. *Chem. Pharm. Bull. (Tokyo)* **34**, 2840-2851 (1986).

11. Zhao X, *et al.* Design and synthesis of pinanamine derivatives as anti-influenza A M2 ion channel inhibitors. *Antiviral Res.* **96**, 91-99 (2012).

12. Yao CH, *et al.* Discovery of novel N-beta-D-xylosylindole derivatives as sodium-dependent glucose cotransporter 2 (SGLT2) inhibitors for the management of hyperglycemia in diabetes. *J. Med. Chem.* **54**, 166-178 (2011).

13. Tsuboi K, Yamai Y, Watanabe H, Kinoshita H. Preparation of N2-[(benzimidazol-2-yl or imidazo[4,5-b]pyridin-2-yl)methyl]amino acid amide derivatives as inhibitors of sensory neuron specific sodium channel (SNS). EPO Patent WO2010074193A1 (2010).

14. Bailey N, *et al.* Novel 1,5-benzodiazepindione gastrin/CCKB antagonists. *Bioorg. Med. Chem. Lett.* **7**, 281-286 (1997).

15. Chuckowree I, *et al.* Synthesis of a 1, 3, 5-benzotriazepine-2, 4-dione based library. *Tetrahedron Lett.* **53**, 3607-3611 (2012).

16. Clegg MA, *et al.* Optimization of Naphthyridones into Selective TATA-Binding Protein Associated Factor 1 (TAF1) Bromodomain Inhibitors. *ACS Med. Chem. Lett.* **12**, 1308-1317 (2021).

17. Hanigan TW, Aboukhatwa SM, Taha TY, Frasor J, Petukhov PA. Divergent JNK Phosphorylation of HDAC3 in Triple-Negative Breast Cancer Cells Determines HDAC Inhibitor Binding and Selectivity. *Cell Chem. Biol.* **24**, 1356-1367 e1358 (2017).

18. Tian W, Chen C, Lei X, Zhao J, Liang J. CASTp 3.0: computed atlas of surface topography of proteins. *Nucleic Acids Res.* **46**, W363-W367 (2018).

19. Dundas J, Ouyang Z, Tseng J, Binkowski A, Turpaz Y, Liang J. CASTp: computed atlas of surface topography of proteins with structural and topographical mapping of functionally annotated residues. *Nucleic Acids Res.* **34**, W116-118 (2006).
