# Supplementary material for: Non-covalent inhibitors of thioredoxin glutathione reductase with schistosomicidal activity in vivo
Source: Nat Commun. 2023 Jun 22;14:3737. doi: 10.1038/s41467-023-39444-y (PMC10287695; doi:10.1038/s41467-023-39444-y)
Supplement: Supplementary file 3 — Description of Additional Supplementary Files [file 41467_2023_39444_MOESM3_ESM.pdf]

Description of Additional Supplementary Files

Filename: Supplementary_movie_1.mp4

Description: An analysis of the free energy minima and maxima for the water probe in subpockets A-C using SZMAP and WaterOrientation extension in VIDA. The movie was generated in PyMOL.

Filename: Supplementary_movie_2.pptx

Description: Phenotypic assessment of NTS viability upon PZQ exposure for 24 hours at indicated concentration.
