# Supplementary material for: Non-covalent inhibitors of thioredoxin glutathione reductase with schistosomicidal activity in vivo
Source: Nat Commun. 2023 Jun 22;14:3737. doi: 10.1038/s41467-023-39444-y (PMC10287695; doi:10.1038/s41467-023-39444-y)
Supplement: Supplementary file 5 — Supplementary Movie 2 [file 41467_2023_39444_MOESM5_ESM.pptx]

## Slide 1
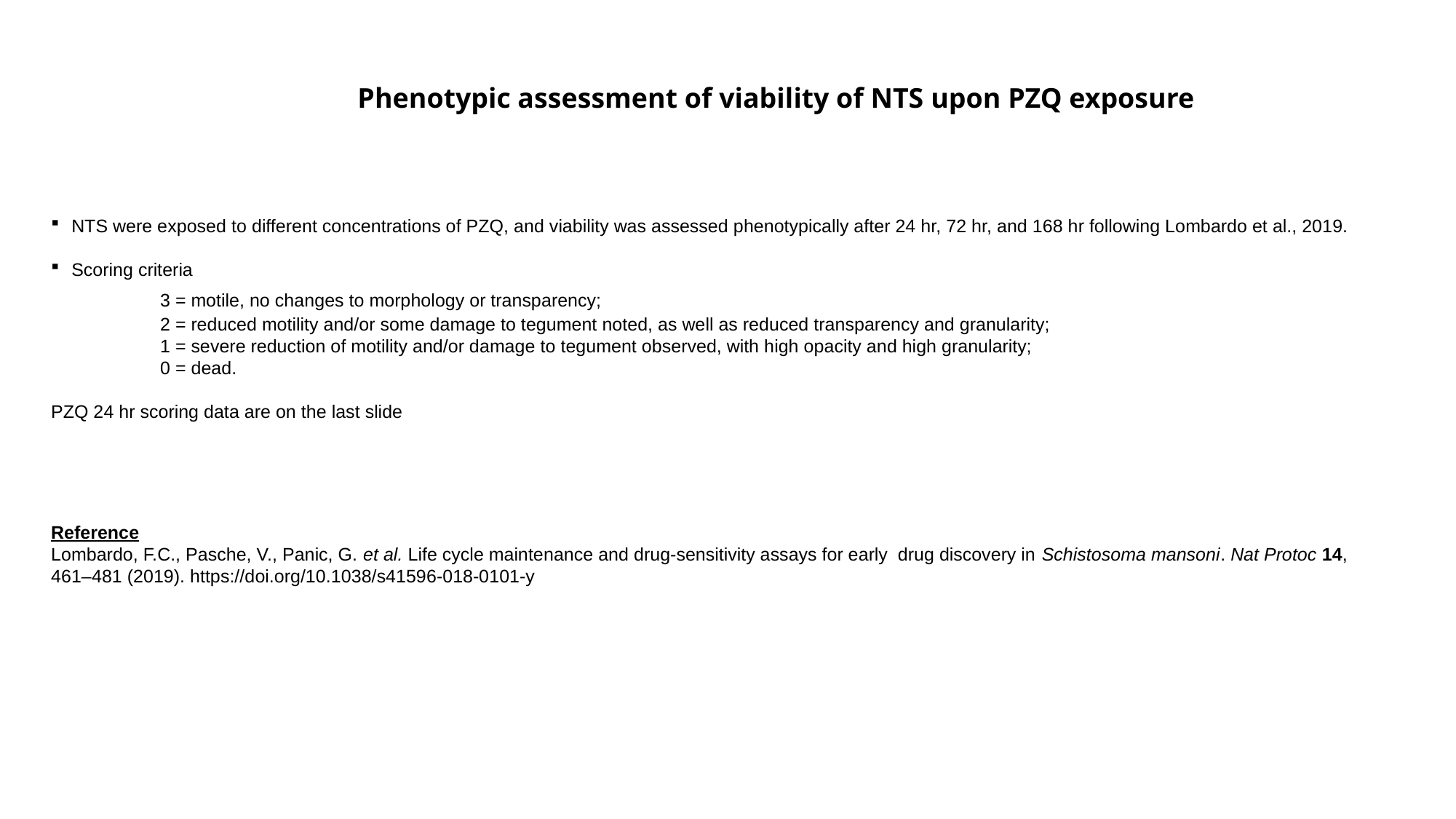

Phenotypic assessment of viability of NTS upon PZQ exposure
NTS were exposed to different concentrations of PZQ, and viability was assessed phenotypically after 24 hr, 72 hr, and 168 hr following Lombardo et al., 2019.
Scoring criteria
	3 = motile, no changes to morphology or transparency;
	2 = reduced motility and/or some damage to tegument noted, as well as reduced transparency and granularity;
	1 = severe reduction of motility and/or damage to tegument observed, with high opacity and high granularity;
	0 = dead.
PZQ 24 hr scoring data are on the last slide
Reference
Lombardo, F.C., Pasche, V., Panic, G. et al. Life cycle maintenance and drug-sensitivity assays for early drug discovery in Schistosoma mansoni. Nat Protoc 14, 461–481 (2019). https://doi.org/10.1038/s41596-018-0101-y

## Slide 2
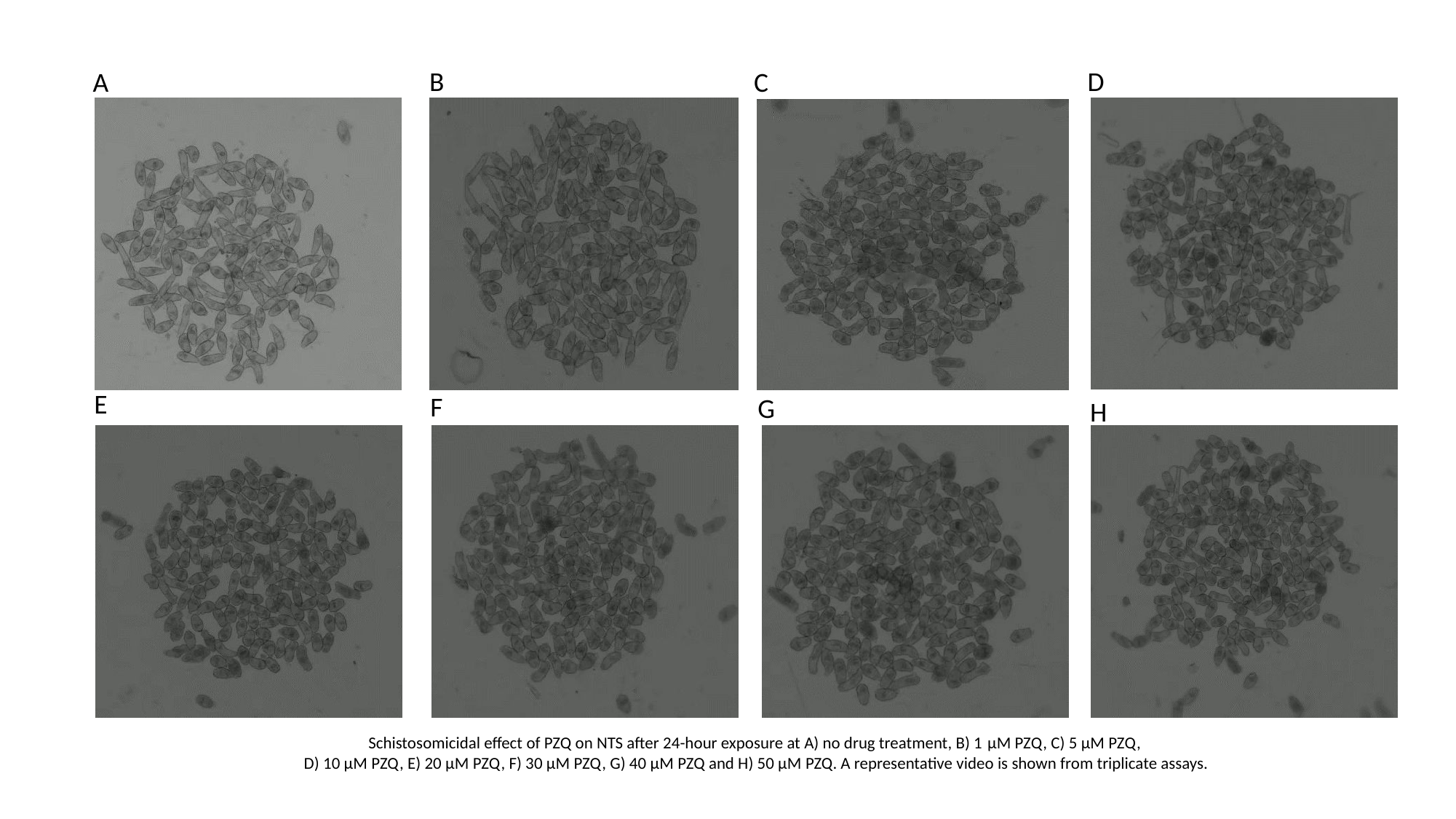

B
D
A
C
E
F
G
H
Schistosomicidal effect of PZQ on NTS after 24-hour exposure at A) no drug treatment, B) 1 µM PZQ, C) 5 µM PZQ,
D) 10 µM PZQ, E) 20 µM PZQ, F) 30 µM PZQ, G) 40 µM PZQ and H) 50 µM PZQ. A representative video is shown from triplicate assays.
