# Supplementary material for: Non-covalent inhibitors of thioredoxin glutathione reductase with schistosomicidal activity in vivo
Source: Nat Commun. 2023 Jun 22;14:3737. doi: 10.1038/s41467-023-39444-y (PMC10287695; doi:10.1038/s41467-023-39444-y)
Supplement: Supplementary file 6 — Reporting Summary [file 41467_2023_39444_MOESM6_ESM.pdf]

nature portfolio

Corresponding author(s): Francesco Angelucci, David Williams, Pavel A. Petukhov

Last updated by author(s): May 30, 2023

Reporting Summary

Nature Portfolio wishes to improve the reproducibility of the work that we publish. This form provides structure for consistency and transparency in reporting. For further information on Nature Portfolio policies, see our Editorial Policies and the Editorial Policy Checklist.

Statistics

For all statistical analyses, confirm that the following items are present in the figure legend, table legend, main text, or Methods section.

n/a	Confirmed	
☐	☒	The exact sample size (*n*) for each experimental group/condition, given as a discrete number and unit of measurement
☐	☒	A statement on whether measurements were taken from distinct samples or whether the same sample was measured repeatedly
☐	☒	The statistical test(s) used AND whether they are one- or two-sided<br>*Only common tests should be described solely by name; describe more complex techniques in the Methods section.*
☐	☒	A description of all covariates tested
☒	☐	A description of any assumptions or corrections, such as tests of normality and adjustment for multiple comparisons
☐	☒	A full description of the statistical parameters including central tendency (e.g. means) or other basic estimates (e.g. regression coefficient) AND variation (e.g. standard deviation) or associated estimates of uncertainty (e.g. confidence intervals)
☐	☒	For null hypothesis testing, the test statistic (e.g. *F*, *t*, *r*) with confidence intervals, effect sizes, degrees of freedom and *P* value noted<br>*Give P values as exact values whenever suitable.*
☒	☐	For Bayesian analysis, information on the choice of priors and Markov chain Monte Carlo settings
☒	☐	For hierarchical and complex designs, identification of the appropriate level for tests and full reporting of outcomes
☒	☐	Estimates of effect sizes (e.g. Cohen's *d*, Pearson's *r*), indicating how they were calculated

Our web collection on statistics for biologists contains articles on many of the points above.

Software and code

Policy information about availability of computer code

Data collection	Smart EPU Software 3.0 was used for cryo-EM data collections.
Data analysis	Relion 3.1 was used to analyse cryo-EM images and to carry out 2D and 3D classifications. Phenix 1.19.2 was used for local anysotropic sharpening and to refine 3D structures derived from cryo-EM data by real-space refinement. Pymol 1.8.2.0, UCSF Chimera 1.16.0, UCSF Chimera X 1.6 and Coot 0.9.8.1 EL were used to make figures and carry out structural analyses. BioRad CFX Maestro 5.2 has been used to analyze TSA data. Computer-based design was facilitated by OpenEye/Cadence software: SZMAP version 1.6.1.0, BROOD version 3.1.4.0, VIDA version 5.0.3, and Chemical Computing Group software: Molecular Operating Environment (MOE), version 2019.01, and Schrödinger software: PyMOL, version 2.5.4.

For manuscripts utilizing custom algorithms or software that are central to the research but not yet described in published literature, software must be made available to editors and reviewers. We strongly encourage code deposition in a community repository (e.g. GitHub). See the Nature Portfolio guidelines for submitting code & software for further information.

Data

Policy information about availability of data

All manuscripts must include a data availability statement. This statement should provide the following information, where applicable:

- Accession codes, unique identifiers, or web links for publicly available datasets
- A description of any restrictions on data availability
- For clinical datasets or third party data, please ensure that the statement adheres to our policy

The cryo-EM data generated in this study have been deposited in the PDB and in the EM data bank under accession codes 8A1R [https://www.rcsb.org/structure/8A1R] and EMD-15084 [https://www.ebi.ac.uk/emdb/EMD-15084], respectively. The cryo-EM data will be released immediately upon publication. The X-ray-derived data of small molecular fragments in complex with TGR used in this study to design the compounds here described are available in the PDB under accession codes 6FTC [https://www.rcsb.org/structure/6FTC], 6FMU [https://www.rcsb.org/structure/6FMU], 6FMZ [https://www.rcsb.org/structure/6FMZ] and 6FP4 [https://www.rcsb.org/structure/6FP4], 2X99 [https://www.rcsb.org/structure/3X99], 2X8G [https://www.rcsb.org/structure/2X8G], 2X8H [https://www.rcsb.org/structure/2X8H], 6ZST [https://www.rcsb.org/structure/6ZST], 6ZP3 [https://www.rcsb.org/structure/6ZP3], 6ZLP [https://www.rcsb.org/structure/6ZLP], 6ZLB [https://www.rcsb.org/structure/6ZLB], 7B02 [https://www.rcsb.org/structure/7B02], 7NPX [https://www.rcsb.org/structure/7NPX], 2V6O [https://www.rcsb.org/structure/2V6O], 6RTJ [https://www.rcsb.org/structure/6RTJ], 6RTO [https://www.rcsb.org/structure/6RTO], 6RTM [https://www.rcsb.org/structure/6RTM], 3H4K [https://www.rcsb.org/structure/3H4K]. The X-ray-derived data of human GR in complex with NAPD+ used in this study to formulate the inhibition mechanism of the compounds depicted in Fig. 5 is available in the PDB under accession codes 3D4K [https://www.rcsb.org/structure/3DK4].
Plasmodium falciparum thioredoxin reductase protein sequence: https://www.ncbi.nlm.nih.gov/protein/CAA60574.1/
Design of inhibitors was facilitated by ChEMBL25 database [http://doi.org/10.6019/CHEMBL.database.25]

Human research participants

Policy information about studies involving human research participants and Sex and Gender in Research.

Reporting on sex and gender	not applicable
Population characteristics	not applicable
Recruitment	not applicable
Ethics oversight	not applicable

Note that full information on the approval of the study protocol must also be provided in the manuscript.

Field-specific reporting

Please select the one below that is the best fit for your research. If you are not sure, read the appropriate sections before making your selection.

☒ Life sciences ☐ Behavioural & social sciences ☐ Ecological, evolutionary & environmental sciences

For a reference copy of the document with all sections, see nature.com/documents/nr-reporting-summary-flat.pdf

Life sciences study design

All studies must disclose on these points even when the disclosure is negative.

Sample size	Sample size calculations for means to obtain statistical power of 80% to detect 90% decrease in worm burden between the treated and the control groups at alpha=0.05, 5 mice per group were required.
Data exclusions	No data excluded.
Replication	All biochemical assays were done in triplicate. Experiments with cultured worms and Vero cells were done in triplicate, on mixed sex worms. In vivo experiments were done one time at the indicated dosage on 5 mice in each treatment and control. Each mouse treated is an experimental replicate. Five mice were used for each treatment to obtain statistical significance due to variability in infection rates. Age- and sex-matched outbred mice were infected with pools of cercariae obtained from >100 infected, outbred snails. This is standard operating procedure in the field. All attempts at replication were successful.
Randomization	Mice were randomly distributed in cages by animal care personnel not involved in the studies. Mice were randomly assigned for the control and experiment groups using a randomization tool embedded in GraphPad Prism.
Blinding	The person administering drugs was not aware of the treatments being given. Assessment of worm burdens was done by a person not administering the treatments. The investigators were blinded by which group of mice received a treatment and the vehicle, as injection was done by technicians from the Comparative Research Center of Rush University Medical Center.

Reporting for specific materials, systems and methods

We require information from authors about some types of materials, experimental systems and methods used in many studies. Here, indicate whether each material, system or method listed is relevant to your study. If you are not sure if a list item applies to your research, read the appropriate section before selecting a response.

Materials & experimental systems

n/a	Involved in the study
☒	☐ Antibodies
☐	☒ Eukaryotic cell lines
☒	☐ Palaeontology and archaeology
☐	☒ Animals and other organisms
☒	☐ Clinical data
☒	☐ Dual use research of concern

Methods

n/a	Involved in the study
☒	☐ ChIP-seq
☒	☐ Flow cytometry
☒	☐ MRI-based neuroimaging

Eukaryotic cell lines

Policy information about cell lines and Sex and Gender in Research

Cell line source(s)	VERO cells were obtained from ATCC.
Authentication	Short tandem repeat profiling was used to authenticate the cell line.
Mycoplasma contamination	Cells were conformed to be mycoplasma-free using a biochemical test kit from Lonza.
Commonly misidentified lines (See ICLAC register)	No commonly misidentified cell lines were used in the study

Animals and other research organisms

Policy information about studies involving animals; ARRIVE guidelines recommended for reporting animal research, and Sex and Gender in Research

Laboratory animals	Mus musculus, Swiss-Webster, female, 8 to 10 weeks old<br>Schistosoma mansoni, NMRI strain, male/female, age not applicable<br>Biomphalaria glabrata snails, strain NMRI, male/female, one month old<br>Schistosoma japonicum, Chinese strain, male/female, age not applicable<br>Oncomelania hupensis hupensis snails, Chinese strain, male/female, one month old<br><br>Mice were housed in 12 h light:dark cycle at 22-25 degrees temperature with relative humidity of 50-70%.
Wild animals	The study did not involve wild animals
Reporting on sex	Only female mice used in the study. Standard practice for maintaining the schistosome lifecycle and drug studies at this stage of development.
Field-collected samples	The study did not involve samples collected from the field
Ethics oversight	This study was approved by the Institutional Animal Care and Use Committee of Rush University Medical Center (20-069; Department of Health and Human Services animal welfare assurance number A-3120–01) and the Institutional Animal Care and Use Committee (IACUC) of the University of Illinois at Chicago (Protocol 19-049).

Note that full information on the approval of the study protocol must also be provided in the manuscript.
